# Archaeal tyrosine recombinases

**DOI:** 10.1093/femsre/fuab004

**Published:** 2021-02-01

**Authors:** Catherine Badel, Violette Da Cunha, Jacques Oberto

**Affiliations:** Université Paris-Saclay, CEA, CNRS, Institute for Integrative Biology of the Cell (I2BC), 91198 Gif-sur-Yvette, France; Université Paris-Saclay, CEA, CNRS, Institute for Integrative Biology of the Cell (I2BC), 91198 Gif-sur-Yvette, France; Université Paris-Saclay, CEA, CNRS, Institute for Integrative Biology of the Cell (I2BC), 91198 Gif-sur-Yvette, France

**Keywords:** tyrosine recombinase, Archaea, mobile genetic element, horizontal transfer, genome evolution, site-specific recombination

## Abstract

The integration of mobile genetic elements into their host chromosome influences the immediate fate of cellular organisms and gradually shapes their evolution. Site-specific recombinases catalyzing this integration have been extensively characterized both in bacteria and eukarya. More recently, a number of reports provided the in-depth characterization of archaeal tyrosine recombinases and highlighted new particular features not observed in the other two domains. In addition to being active in extreme environments, archaeal integrases catalyze reactions beyond site-specific recombination. Some of these integrases can catalyze low-sequence specificity recombination reactions with the same outcome as homologous recombination events generating deep rearrangements of their host genome. A large proportion of archaeal integrases are termed suicidal due to the presence of a specific recombination target within their own gene. The paradoxical maintenance of integrases that disrupt their gene upon integration implies novel mechanisms for their evolution. In this review, we assess the diversity of the archaeal tyrosine recombinases using a phylogenomic analysis based on an exhaustive similarity network. We outline the biochemical, ecological and evolutionary properties of these enzymes in the context of the families we identified and emphasize similarities and differences between archaeal recombinases and their bacterial and eukaryal counterparts.

## Teaser

A number of new archaeal tyrosine recombinases have been reported to catalyze canonical DNA integration, excision, inversion and also reactions beyond site-specific recombination, prompting us to review their properties and analyze their phylogenomic relationships with other known recombinases.

## INTRODUCTION

Recombination of DNA is an essential mechanism ensuring the maintenance, propagation and evolution of genetic information in all living organisms. Homologous recombination is complex, requires energy, involves a number of protein complexes and operates over large regions sharing extensive sequence identity (Sung and Klein 2006; Sun *et al*. 2020). The exchange point can occur anywhere between these regions. Site-specific recombination is an energy-independent process catalyzed by DNA transaction proteins whose primary function is to specifically recognize and recombine two short DNA duplexes sharing some degree of sequence identity (Craig 1988; Dorman and Bogue 2016). In this case, the breakage and joining of DNA requires a particular catalytic amino acid forming a transient covalent bond between the protein and the DNA substrate (Pargellis *et al*. 1988; Smith *et al*. 2004). Conservative site-specific recombinases are classified into two unrelated families, serine and tyrosine recombinases, referring to this catalytic residue (Grindley, Whiteson and Rice 2006; Stark 2014). The recombination promoted by these enzymes is conservative since strand exchange occurs in a precise location of short sequence identity without loss or addition of DNA. Non-conservative site-specific recombinases such as DDE transposases do not depend on sequence identity in the target sequences and require DNA synthesis. Site-specific recombinases are encountered in the three domains of life and have been characterized extensively in both bacteria and eukarya where they perform a number of biological functions such as integration and excision of viral DNA into the host chromosome (Landy 2015), phage resolution (Van Duyne 2015; Meinke *et al*. 2016), plasmid flipping (Jayaram *et al*. 2015), resolution of chromosome dimers (Castillo, Benmohamed and Szatmari 2017), integron shuffling (Escudero *et al*. 2015; Engelstadter, Harms and Johnsen 2016), insertion sequence (IS) element transposition (Siguier *et al*. 2015; Arinkin, Smyshlyaev and Barabas 2019) and induction of gene expression by phase variation (Bayliss 2009). The usefulness of tyrosine recombinases as DNA transaction tools has emerged in a wide range of biotechnological and medical applications (Jayaram *et al*. 2015; Van Duyne 2015; Meinke *et al*. 2016).

In archaea, serine recombinases and DDE recombinases have been observed in transposons but were never fully investigated (Filee, Siguier and Chandler 2007; Krupovic *et al*. 2019). On the other hand, the activities of several archaeal tyrosine recombinases have been analyzed, reviewed and ranked into two classes (She, Brugger and Chen 2002; She, Chen and Chen 2004). Class I corresponds to the SSV-like integrases found in *Sulfolobales* viruses and whose gene is fragmented upon integration. Class II groups the pNOB8 plasmid-like integrases that follow the phage λ integration paradigm. Additionally, XerA resolvases are encoded by archaeal chromosomes. In the recent years, the study of archaeal tyrosine recombinases has generated considerable experimental data.

In the present review, we discuss archaeal integrases from biochemical, ecological and evolutionary points of view. After a brief overview of the historical context of the discovery of archaeal integrases, we present the insights gained from their sequences and the current knowledge about their mechanisms and recombination target. These aspects are put into the perspective of a systematic phylogenomic analysis. Because the recombination reaction catalyzed by integrases is central to mobile genetic element (MGE) lifestyles, we then highlight some ecological consideration related to archaeal integrases. Finally, we describe recent findings on integrase evolution and genome evolution mediated by integrases. The deep comparison of all available archaeal tyrosine recombinases presented here allows the ranking of these enzymes in defined families and to underline functional similarities and differences with known bacterial and eukaryal recombinases.

## FIRST ACCOUNTS OF INTEGRASE-PROMOTED SITE-SPECIFIC RECOMBINATION

**Figure 1.**
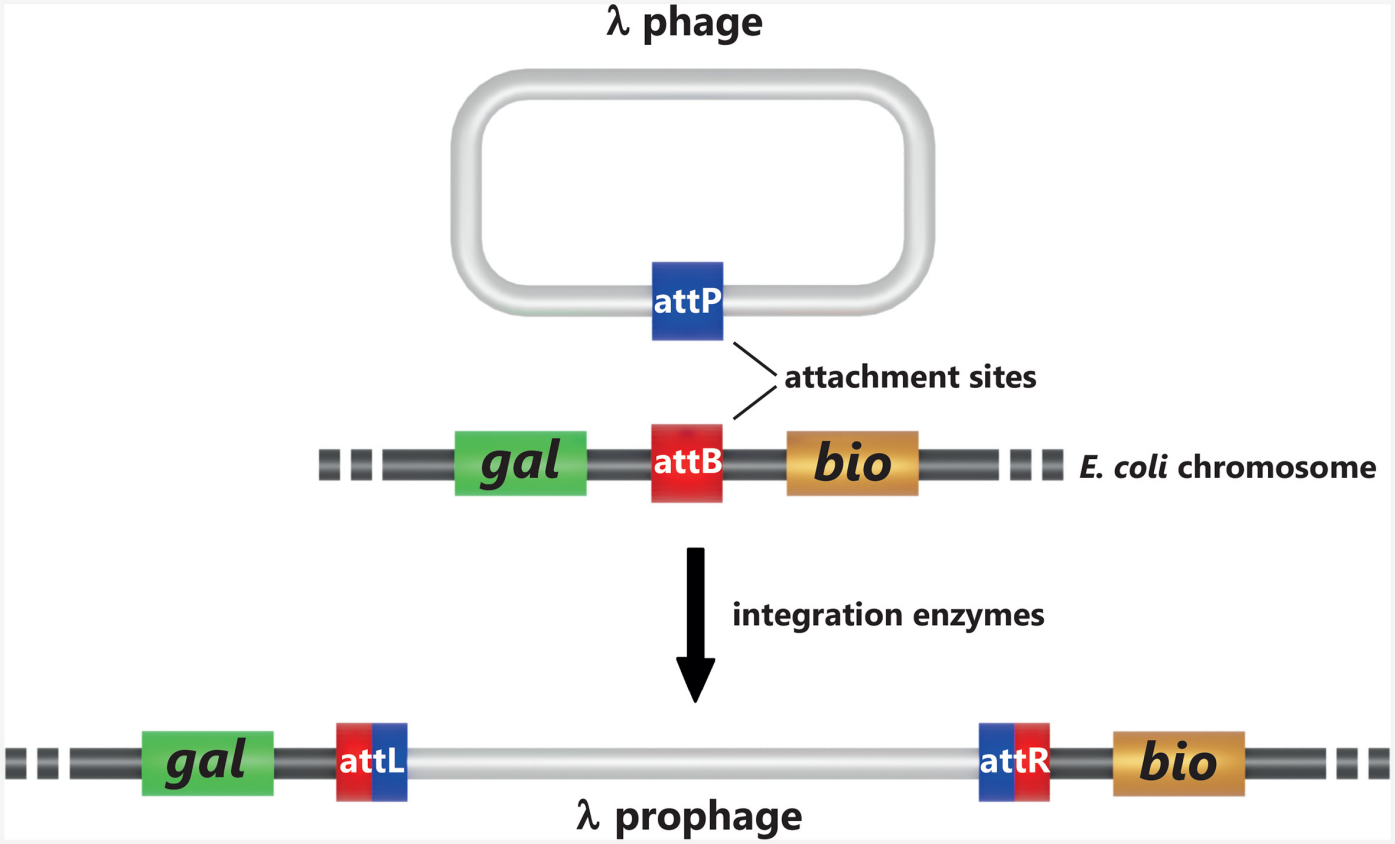
Campbell model for phage λ site-specific recombination. The model presented by Campbell (1963) suggested for the first time the breaking and rejoining of DNA sequences by integration enzymes in order to allow phage λ lysogenization in *Escherichia coli*.

Early investigations reported the discovery of an ultra-microscopic virus capable of either modifying bacteria or destroying them (Twort 1915). A similar virus able to lyse *Shigella* was later isolated and called bacteriophage (d'Hérelle 1917). Bordet and Ciuca defined these bacteria modified by bacteriophages as lysogens since they carried the potential to lyse other cells (Bordet and Ciuca 1920). With the advent of phage genetics, the mechanisms of lytic cycle and lysogeny later became understandable in molecular terms. Building upon his hypothesis of a small region of homology between phage and host chromosome where recombination would take place, Campbell elaborated the foundations of the site-specific recombination pathway and illustrated how bacteriophage λ and other episomes can integrate into, or excise from, bacterial chromosomes (Campbell 1963) (Fig. 1). Zissler isolated the first phage λ mutants unable to lysogenize and interpreted them as lacking a region necessary for integration in the host chromosome that he called *int* (for integration deficient) (Zissler 1967). Simultaneously, the requirement of an enzymatic activity for site-specific recombination was reported for bacteriophage φ80 (Signer and Beckwith 1966). The product of the *int* gene along with the newly identified attachment sites attϕ (attachment φ80) and attB (attachment bacteria) was used to demonstrate site-specific integration by formal genetics (Weil and Signer 1968). Ausubel performed the first radiochemical purification of this enzyme that he called integrase, using phage λ-infected *Escherichia coli* cells (Ausubel 1974). Concomitantly, Nash also purified the Int protein (Nash 1974) that he later used to demonstrate integrative recombination activity *in vitro* (Nash 1975). Remarkably, this relentless experimentation extending over six decades succeeded in identifying all components involved of site-specific recombination at the advent of molecular biology and before DNA sequencing.

### Three types of archaeal tyrosine recombinases

**Figure 2.**
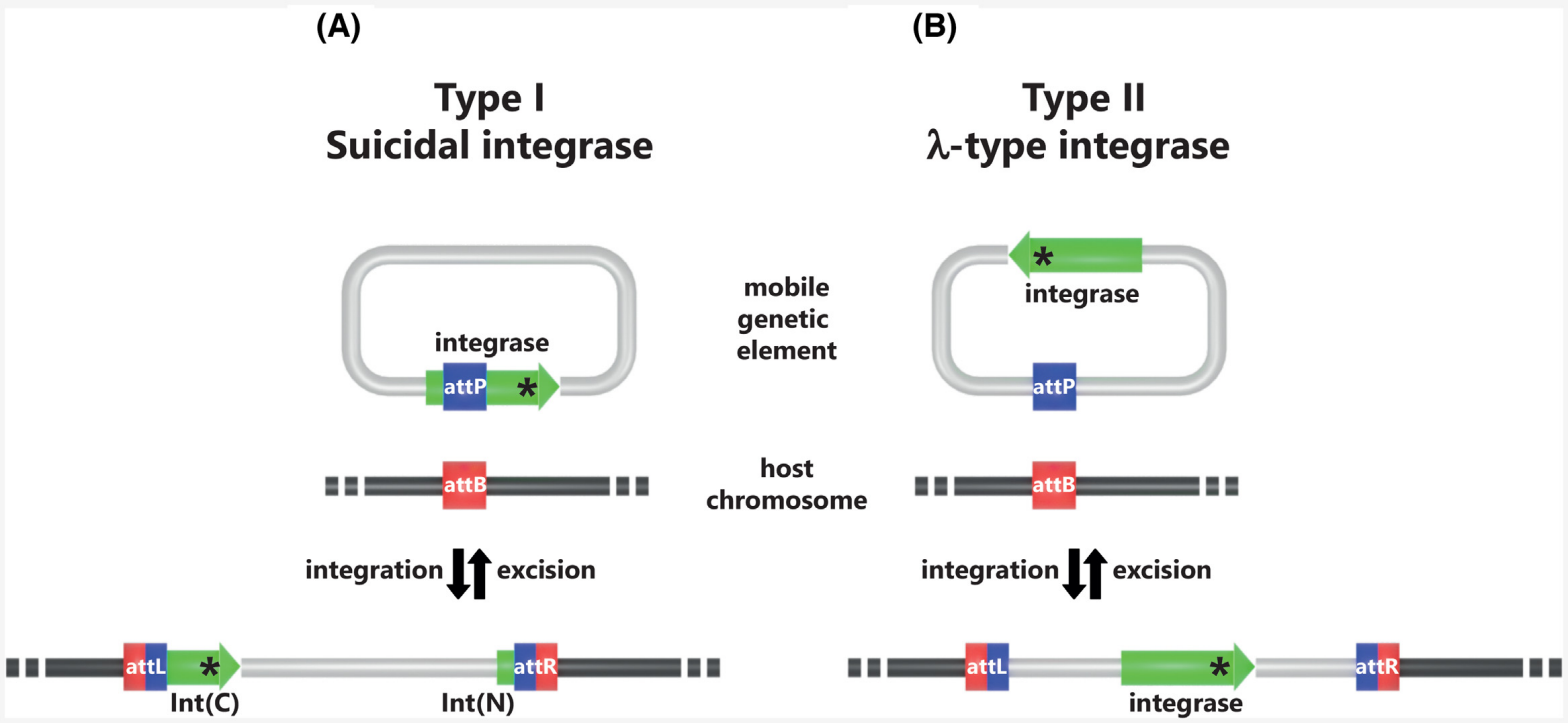
Classes of archaeal integrases. She, Brugger and Chen (2002) proposed the ranking of archaeal integrases into two distinct types: Type I **(A)** and Type II **(B)**. The blue and red squares correspond to the specific recombination sites.

The archaea, which form the third domain of life, often carry extrachromosomal elements some of which were found integrated into the genome. The first report described the presence of a freely replicating and chromosome-integrated element of 15 kb, later called *Sulfolobus* spindle-shaped virus 1 or SSV1 (Schleper, Kubo and Zillig 1992), in the hyperthermophilic *Sulfolobus shibatae* (Grogan, Palm and Zillig 1990). Soon other elements were discovered such as SAV1 (Martin *et al*. 1984), pQX1 (Peng *et al*. 2000), XQ2 (She *et al*. 2001a) and SSV2 (Stedman *et al*. 2003) that could integrate at specific chromosomal loci in various *Sulfolobus* species of the *Crenarchaeota* phylum. SSV1 and SAV1 were shown to form virus-like particles upon UV induction (Martin *et al*. 1984; Frols *et al*. 2007) and turned out to contain identical genomes (Stedman *et al*. 2003). Interestingly, the SSV-type integrases encoded by SSV1, SSV2, pQX1 and XQ2 carry the DNA recombination site within their own gene that would become fragmented upon chromosomal integration, therefore precluding integrase-mediated excision in the absence of an intact gene (She *et al*. 2001a) (Fig. 2A). More recently, SSV-type integrases encoded by *Thermococcus nautili* plasmid pTN3 (Oberto *et al*. 2014; Cossu *et al*. 2017) and by *Thermococcus*sp. 26-2 plasmid pT26-2 (Badel *et al*. 2020) were uncovered in the *Euryarchaeota* phylum as well. Remarkably, recombinases that disrupt their own gene are found exclusively in the archaeal domain and were named suicidal integrases (Badel *et al*. 2020). It would be logical to assume such MGEs would remain irreversibly integrated into their host genome. However, this is not the case. This aspect and the evolutionary implications of suicidal integrases will be discussed below.

The second type of archaeal integrases follows the more classical bacteriophage λ paradigm and maintains an intact gene after integration (Fig. 2B). *Sulfolobus* conjugative plasmid pNOB8 (She *et al*. 1998) encoded such a recombinase allowing to integrate into the genome of *Sulfolobus tokodaii* and several other species (She, Chen and Chen 2004). A systematic genomic search for sequences encoding the more conserved catalytic domain identified a number of archaeal integrases of this type that could be ranked in five families (She, Brugger and Chen 2002).

In addition to the first two types of tyrosine recombinases that correspond to *bona fide* integrases, the majority archaeal genomes possess the XerA (also named XerC) site-specific recombinase that, similarly as the bacterial XerC/D homologs, resolves chromosome dimers occurring during DNA replication (Cortez *et al*. 2010). The major difference between bacteria and archaea is that the latter lack an FtsK homolog, which is essential in bacterial systems to displace the reaction equilibrium toward resolution (Bigot *et al*. 2004). This observation suggests that the regulation of archaeal chromosome dimer resolution operates with a different mechanism than in bacteria.

## DEFINITION OF THE FAMILY OF TYROSINE RECOMBINASES

### Insights from primary sequences

The development of sequence databases leads to the detection of novel enzymes related to phage λ integrase and the identification of their functional domains by comparative analysis. Despite distant relationships in protein primary sequences, all bacteriophage site-specific recombinase C-terminal ends could be aligned with the yeast 2µ plasmid FLP protein (Argos *et al*. 1986). In particular, the perfect conservation of the three residues (HXXR…Y) highlighted a potential active site. The presence of a tyrosine in that region suggested a catalytic function for this residue in DNA cleavage (Argos *et al*. 1986). A subsequent analysis revealed yet another conserved arginine residue upstream leading to the consensus tetrad R…HXXR…Y (Abremski and Hoess 1992). These enzymes defined a new family of proteins, the tyrosine recombinases. Afterward, the alignment of all the tyrosine recombinases available in the databases underlined an important sequence diversity among these enzymes and the conservation of important residues composing the catalytic domain that spans ∼180 residues as confirmed by mutational analysis (Esposito and Scocca 1997; Nunes-Duby *et al*. 1998).

**Figure 3.**
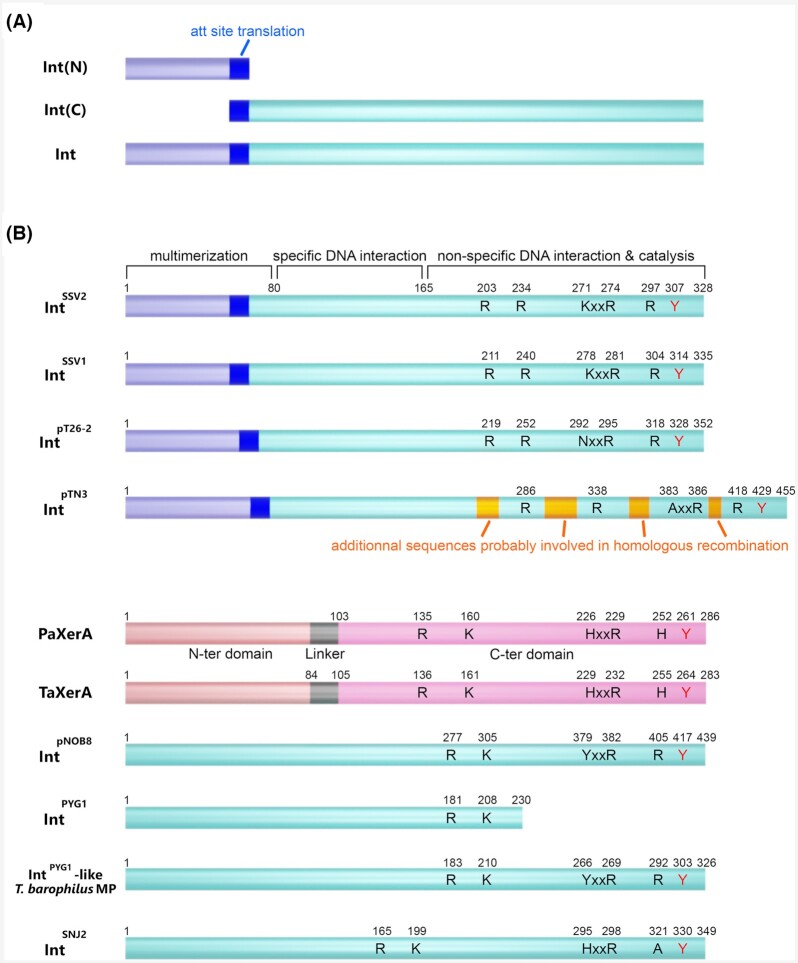
Archaeal integrases sequence domains and conserved residues.**(A)** Suicidal integrases are either encoded by their intact gene or the regions corresponding to the N-terminal portion or Int(N), and C-terminal region or Int(C) are separated upon MGE integration. **(B)** The conserved catalytic residues are indicated for all characterized archaeal tyrosine integrases from Table 1. The domains of particular interest are indicated. Functional domains were dissected for IntSSV2 (Zhan, Zhou and Huang 2015). IntpTN3 presents additional loop that may be responsible for its unprecedented dual catalytic activity (Cossu *et al*. 2017). PaXerA and TaXerA structure was resolved and corresponds to two domains separated by a linker (Serre *et al*. 2013; Jo *et al*. 2016).

The study of archaeal organisms also revealed integrase-encoding genes from *Sulfolobus shibatae* spindle-shaped virus (SSV1) (Palm *et al*. 1991) that split upon integration (Fig. 3A) and from *Sulfolobus* NOB8-H2 pNOB8 plasmid (She *et al*. 1998). On the basis of a slightly divergent consensus, two subfamilies were identified: the SSV1-type integrases (R…KXXR…Y) and the pNOB8-type integrases (R…YXXR…Y) (She, Chen and Chen 2004) (Fig. 3B). IntSSV1 and XerA from *Pyrococcus abyssi* (PaXerA) were shown to form a covalent intermediate with the substrate DNA and the implication of the tyrosine Y314 was evidenced for IntSSV1 (Serre *et al*. 2002, 2013; Zhan *et al*. 2012). A substitution of this tyrosine abolished IntSSV1 substrate cleavage activity confirming its importance for catalysis in archaea (Letzelter, Duguet and Serre 2004). Similarly to previously characterized tyrosine recombinases, the active residues are localized at the C-terminal end of the protein and are involved in DNA cleavage and ligation catalysis (Zhan, Zhou and Huang 2015) (Fig. 3B).

The highly divergent N-terminal regions of tyrosine recombinases suggested early on their involvement in features unique to each system such as specific sequence recognition (Argos *et al*. 1986). This diversity also reflected the fact that some recombinases such as phage integrases would recognize two distinct DNA sites instead of a single one (Esposito and Scocca 1997). It can be assumed that archaeal recombinases use their N-terminal regions to recognize and bind to their specific site. Gel retardation experiments with truncated forms of IntSSV1 indicated that in addition to the full-length protein, both the first half (N175) and the second half (C174) would bind to the specific DNA target (Zhan *et al*. 2012). A similar approach using *Sulfolobus islandicus* IntSSV2 demonstrated that the N-terminal extremity controls multimerization whereas the middle portion officiates in the specific DNA interaction (Zhan, Zhou and Huang 2015).

### Extending the archaeal tyrosine recombinase diversity

Archaeal tyrosine recombinases belong to the NCBI DNA_BRE_C superfamily (cl00213 or cd00397) that groups the DNA breaking–rejoining enzymes with a catalytic domain in C-terminal position. In addition to tyrosine site-specific recombinases such as integrases, this superfamily also includes Type IB topoisomerases, as they share conserved active site residues and the same fold in their catalytic domain (Cheng *et al*. 1998). This DNA_BRE_C superfamily is composed of five major Pfam domains: Phage_integrase (PF00589), Phage_integr_3 (PF16795), Topoisom_I (PF01028), DUF3504 (PF12012) and Integrase_1 (PF12835). The DUF3504 and Integrase_1 domains are specific to eukarya and bacteria, respectively.

In order to propose a comprehensive overview of archaeal integrase diversity, we undertook a phylogenomic analysis based on an exhaustive similarity network to assign all these enzymes to their respective families. This classification also illustrates the phylogenetic relationships between the families and enlightens the evolutionary links between the three domains of life. For this deep investigation, we extracted from the conserved domain database of the NCBI all archaeal protein sequences belonging to the cl00213 superfamily. To this dataset, we added the previously reported integrases encoded by archaeal viruses (Pauly *et al*. 2019) and the reconstructed suicidal integrases from the pTN3 and pT26-2 plasmid families (Cossu *et al*. 2017; Badel *et al*. 2019, 2020). Partial sequences <150 residues were eliminated from the initial dataset and redundancy was reduced using the UCLUST program (Edgar 2010) to eliminate sequences sharing >90% identity (Fig. S1, Supporting Information). Due to the reported high divergence in tyrosine recombinase sequences especially in the N-terminal region, molecular phylogeny tools are ill- adapted to infer the evolutionary relationships between the members of this superfamily (Esposito and Scocca 1997). In a previous report, we successfully used a different methodology based on protein similarity networks to establish relationships between different archaeal integrase families (Badel *et al*. 2020). A similar approach was conducted on the present archaeal integrase dataset using the SiLiX program (Miele, Penel and Duret 2011) in order to define families sharing 25% identity covering at least 60% of the protein.

**Figure 4.**
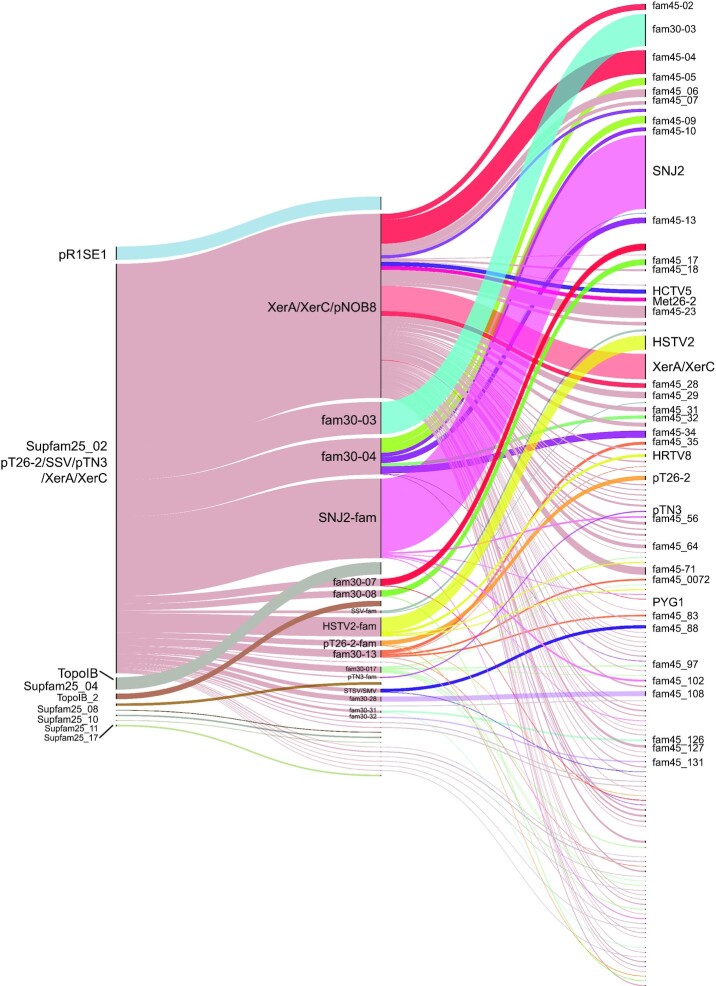
Classification of archaeal tyrosine recombinases. Alluvial diagram showing the integrase distribution across the different archaeal tyrosine recombinase superfamilies (left), families (center) and subfamilies (right), predicted based on the conservation identity threshold of 25%, 30% and 45%, respectively, using SiLiX (Miele, Penel and Duret 2011). In this diagram, blocks represent clusters of proteins and stream fields between the blocks represent changes in clustering attribution of these proteins to superfamily, family and subfamily over the selected identity threshold. Block height is proportional to the size of the protein cluster and the height of a stream field is proportional to the number of proteins contained within the blocks connected by the stream field. Our classification retained the 17 families containing >13 members. The graph was drawn using RawGraphs (https://app.rawgraphs.io) to map data dimensions onto visual variables. The raw data are available in Table S1 (Supporting Information).

Among the 4341 archaeal sequences of the cl00213 superfamily, 93.6% were distributed into three major groups (Fig. 4; Table S1, Supporting Information). The first group, tentatively named pR1SE1, contains integrases encoded by *Haloarchaea* and their MGEs such as the pR1SE plasmid. The second group TopoIB corresponds to the eukaryotic-like topoisomerase I (TopoIB) encoded mostly by *Thaumarchaea*, *Bathyarchaea* and *Aigarchaea*. Our results indicated that no archaeal integrases belong to the group of eukaryotic-like topoisomerases IB, even though a conserved structure between eukaryotic topoisomerase I and the catalytic domain bacterial tyrosine recombinases was reported (Cheng *et al*. 1998). The absence of overall amino acid conservation between these enzymes (Cheng *et al*. 1998) further highlights the divergence we observed. The third group Supfam25-02 is the largest comprising over 89% of the total sequences. It encompasses the characterized archaeal XerA recombinases and integrases identified in several MGE families such as pNOB8, pT26-2, Met26-2, SNJ2, pTN3 and SSV (She, Brugger and Chen 2002; Liu *et al*. 2015; Cossu *et al*. 2017; Wang *et al*. 2018; Badel *et al*. 2020). A similar large clade of archaeal integrases from pNOB8, pT26-2, SNJ2, pTN3 and SSV was previously observed by phylogenetic analysis (Wang *et al*. 2018). Using a slightly more stringent criterion (30% identity covering 60% of the protein), we could rank these sequences into the previously well-characterized archaeal integrases families named after their respective representative SSV, pTN3, pT26-2, SNJ2 and pNOB8. This criterion was instrumental in ranking all archaeal tyrosine recombinases into 17 major families, including newly identified integrases families such as HSTV2, STSV/SMV, fam30-07 and fam30-04 (Fig. 4; Table S1, Supporting Information).

**Figure 5.**
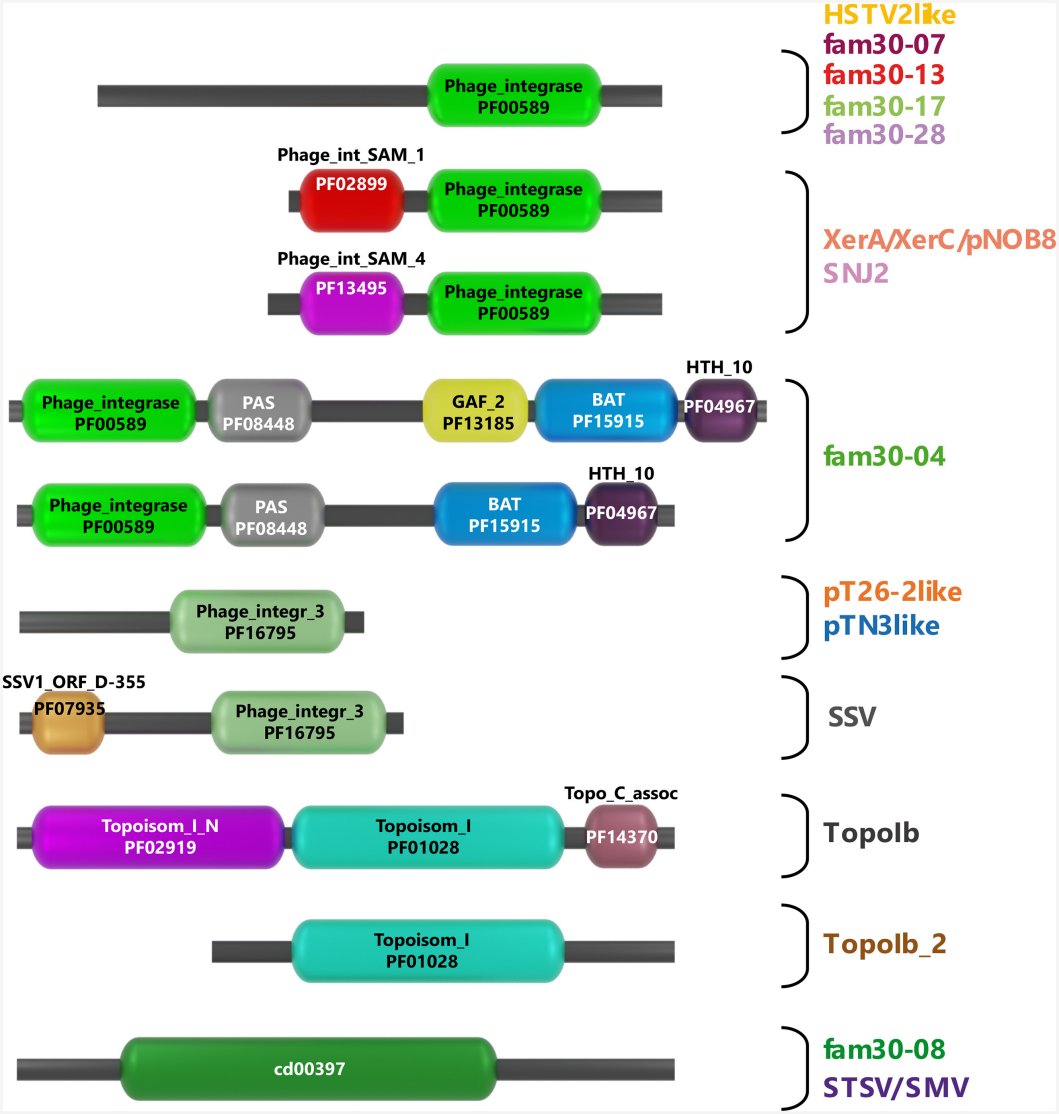
Pfam domain combinations in the 17 major tyrosine recombinase families. The most frequent combinations of Pfam domains for each major tyrosine recombinase are represented. No Pfam could be detected for the fam30-08 and STSV/SMV families even if both belong to the cd00397 superfamily. The result of the conserved domain search is available in Table S1 (Supporting Information).

**Figure 6.**
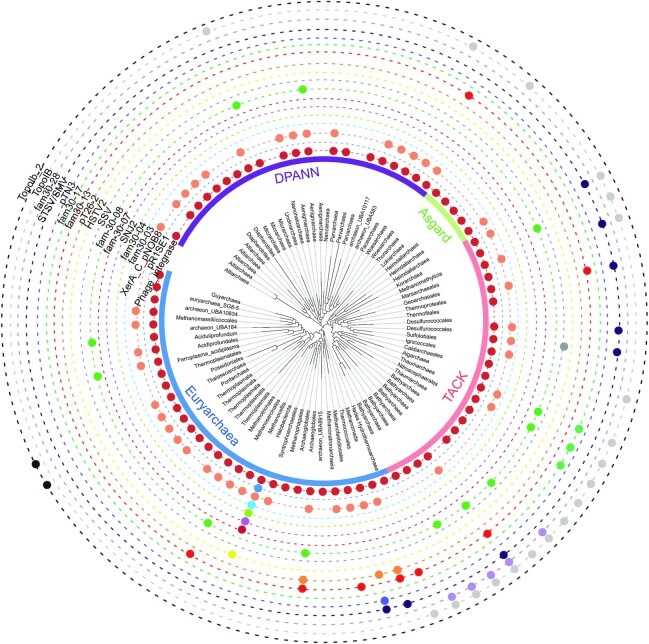
Distribution of the 17 major tyrosine recombinase families among the archaeal diversity. Each tyrosine recombinase family corresponds to a dot. In order to visualize the distribution of the major tyrosine recombinase families on the archaeal diversity, we used data from AnnoTree (Mendler *et al*. 2019) with additional manual curation. The archaeal tree was obtained from the Genome Taxonomy Database (GTDB Release 03-RS86) (Parks *et al*. 2020), generated from 122 core proteins and exported using taxonomic orders as resolution level. The presence/absence profiles for each family were visualized using iTOL (Letunic and Bork 2019).

In order to test whether these connections reflected the conservation of existing domains, we scanned each protein for Pfam domains using the Conserved Domain CD-Search with an *e*-value of 1e-05 (Marchler-Bauer and Bryant 2004) (Fig. S1 and Table S1, Supporting Information). We observed that the different families corresponded mainly to different combinations of Pfam domains, or yet unknown domains. The pTN3like, pT26-2like and SSV corresponded to suicidal integrases with the Phage_integr_3 domain (PF16795). Our classification suggests that suicidal integrases correspond to a recent adaptation originating from a single event within the monophyletic Superfamily 25_02 (Figs 4 and 5) in agreement with previous phylogenetic and network analyses (Wang *et al*. 2018; Badel *et al*. 2019). The TopoIb and TopoIb_2 families both contained the Topoisom_I central domain (PF01028). The TopoIb_2 family is related to viral/bacterial origin whereas the TopoIb family comprises the additional eukaryotic domains Topoisom_I_N (PF02919) Topo_C_assoc (PF14370) (Brochier-Armanet, Gribaldo and Forterre 2008). The major families XerA/XerC/pNOB8 or SNJ2 corresponded to proteins with both Phage_int_SAM (PF02899, PF13495) associated to the Phage_integrase domain (PF00589). In the families HSTV2like, fam30-07, fam30-07, fam30-17 and fam30-28 we detected the single Phage_integrase domain (PF00589). A particular family, fam30-04, composed of larger proteins carried the Phage_integrase (PF00589) BAT (PF15915) and HTH_10 (PF04967) domains with some of them also containing the GAF_2 domain (PF13185). We did not detect known Pfam domains in the fam30-08 and STSV/SMV families. Overall, the diversity of archaeal tyrosine recombinases is explained by the highly variable domain composition of the 17 major families as they only share a single Pfam domain, Phage_integrase (PF00589) (Figs 5 and 6). Archaeal integrases are not equally distributed among archaeal species, as for example the pR1SE, HSTV2 and SNJ2 families are restricted to Halobacteria and their MGEs (Wang *et al*. 2018). Our analysis confirmed this limited distribution, while other integrase families are spread among different classes or phyla such as XerA/XerC/pNOB8 and fam30-08. Further phylogenomic studies will be required to understand how horizontal gene transfer generated the patchy distribution of tyrosine recombinases among archaeal phyla.

**Figure 7.**
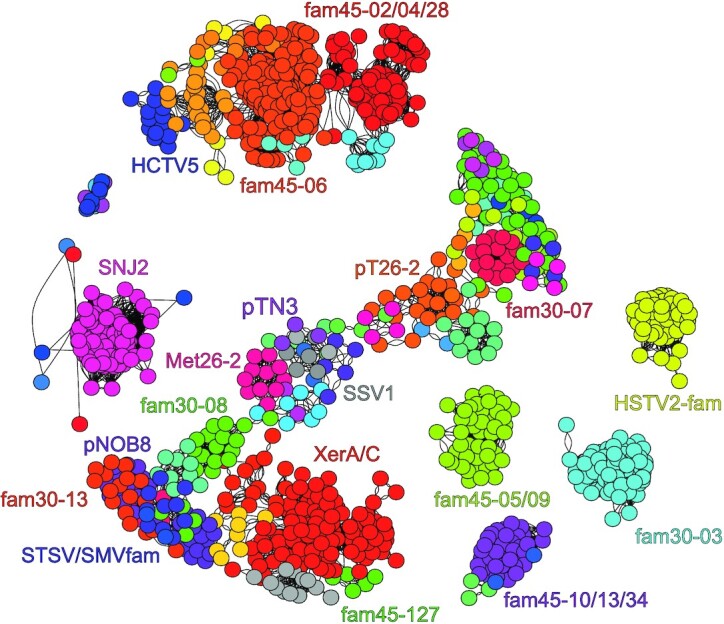
Similarity network of archaeal tyrosine recombinases. A similarity network performed with SiLiX (Miele, Penel and Duret 2011) and visualized with Igraph (https://igraph.org) was used to assign tyrosine recombinases from the superfamily Superfam25-02 to families and subfamilies. The similarities of all against all proteins of the dataset were assessed using BlastP (expect >0.001, with an identity threshold of 30% among 60% of the protein). Protein clustering was achieved by a random walk algorithm. Each circle corresponds to an individual protein colored according to the clustering.

A first observation of our network map (Fig. 7) indicated that all archaeal tyrosine recombinases are not connected through a single network, therefore highlighting the inability to perform a robust phylogeny on this dataset. Early reports already discussed the difficulty to compare the primary sequence of archaeal, bacterial and eukaryotic tyrosine recombinases due to the presence of large portions devoid of detectable homology (Esposito and Scocca 1997; Nunes-Duby *et al*. 1998). A number of recombinases diverged greatly from the main group (Esposito and Scocca 1997) and many singleton integrases could not be ranked in any subfamily (Williams 2002). Our network analysis also confirms two previously reported evolutionary relationships, the first connecting the SSV, pTN3 and pT26-2 integrases families and the second connecting the XerA/C recombinases and the SNJ2, Met26-2 and pNOB8 integrase subfamilies (Wang *et al*. 2018; Badel *et al*. 2019) (Fig. 7).

In order to verify established relationships between tyrosine recombinases in the three domains of life, we extended our network analysis with well-studied bacterial and eukaryotic enzymes from previous reviews (Esposito and Scocca 1997; Nunes-Duby *et al*. 1998). We were able to underline a close relationship between most archaeal tyrosine recombinases and bacterial XerC/D chromosomal resolvases (Colloms *et al*. 1990; Blakely *et al*. 1993), Fim tyrosine-integrase (McCusker, Turner and Dorman 2008), TnpA from transposon Tn554 (Bastos and Murphy 1988), conjugative element ICEBs1 integrase (Suzuki *et al*. 2020), P2 phage integrase (Nilsson *et al*. 2011), virophage integrase (La Scola *et al*. 2008) and Integron_Int (Nunes-Duby *et al*. 1998; Demarre *et al*. 2007). In agreement with previous observations by She *et al*. (She, Brugger and Chen 2002), we confirmed that most archaeal integrases belong to the same superfamily, together with many families of bacterial integrases (Fig. S2 and Table S2, Supporting Information). On the other hand, no direct relationship was observed between archaeal tyrosine recombinases and P1 phage Cre, λ phage Int, yeast 2µ plasmid FLP, DusA-associated integrases (DAI) (Farrugia *et al*. 2015), plasmids R64 shufflon-specific DNA recombinase Rci (Kubo, Kusukawa and Komano 1988) and Tn916 tyrosine recombinases (Lu and Churchward 1994) (Fig. S2 and Table S2, Supporting Information). Interestingly, we did not observe the clade formed by the archaeal integrases from the BJ1 and phiCh1/HCTV-5 viruses (Atanasova *et al*. 2012), P1 Cre and 2µ FLP that was reported by Wang *et al*. (2018). Considering that FLP was previously reported as one the tyrosine recombinases that had greatly diverged (Esposito and Scocca 1997), this discrepancy might be attributed to long branch attraction issues in Wang *et al*. phylogenetic analysis, an artifact to which network analysis is immune. Remarkably, the pR1SE1 archaeal integrase family (Erdmann *et al*. 2017) could not be connected to any of the other tested tyrosine recombinase families.

We have observed many integrase families that do not share relationships with any other known family (Fig. 4; Table S1, Supporting Information). Among these, 27 families harbor <7 members and are encoded, for example, by well-studied viruses such as the *Sulfolobus* turreted icosahedral viruses 1 and 2 (STIV1 and STIV2) (Rice *et al*. 2004; Happonen *et al*. 2010) or by the uncharacterized *Methanocaldococcus*sp. FS406.22 plasmid pFS01 (METSF_2, Joint Genome Institute, unpublished). In addition, we witnessed up to 104 individual integrases not ranked in any family and constituting as many shadow areas remaining to be explored. With the increase in available sequences, we anticipate further analyses able to establish phylogenetic relationships between these tyrosine recombinases and identify new conserved residues conveying additional functions.

### Insights from tertiary structures

**Figure 8.**
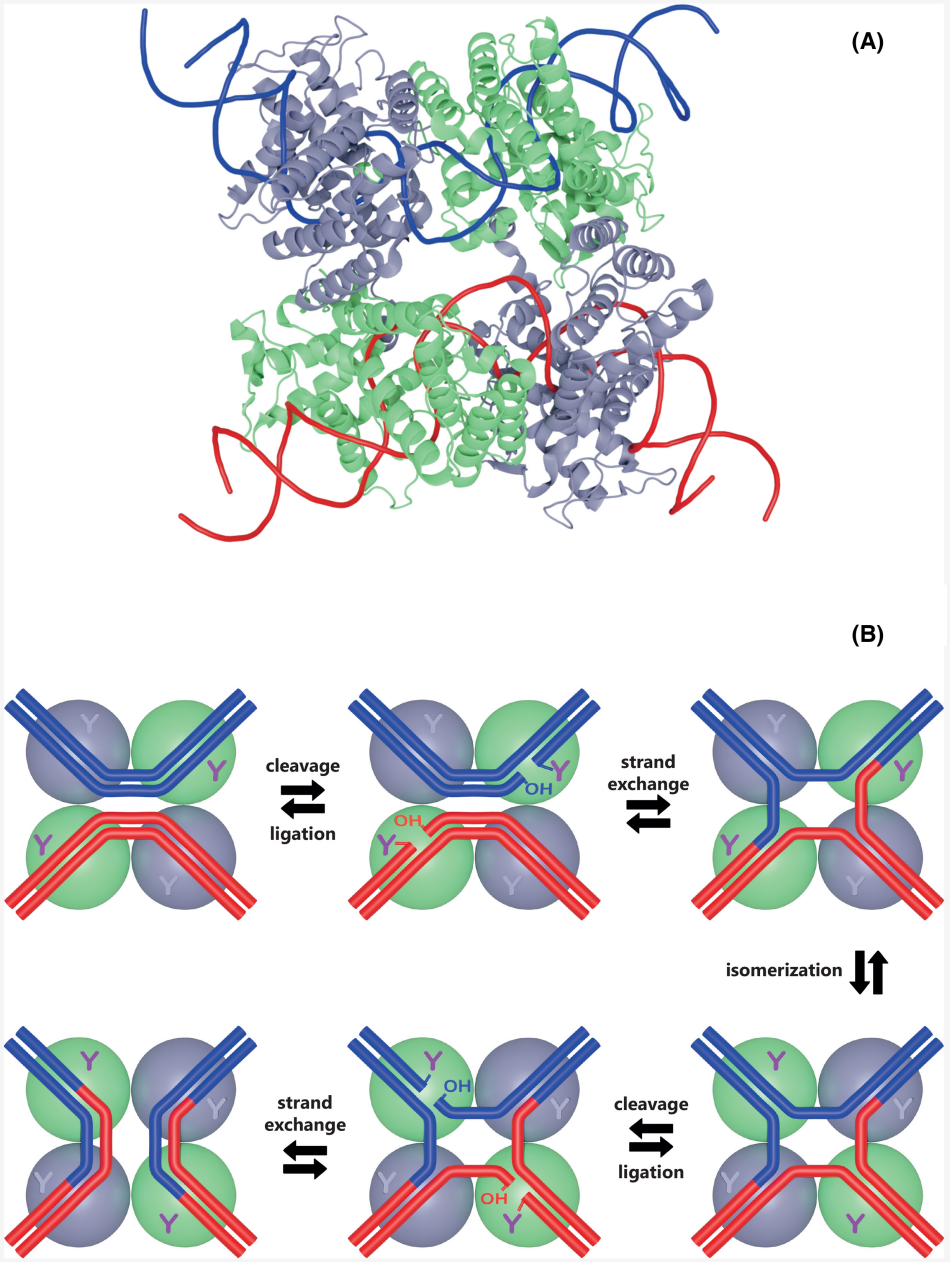
Tyrosine recombinase model and site-specific recombination reaction. **(A)** Structural model of the Cre-loxA pre-synapse complex. The tetrameric conformation of the Cre-loxA pre-synapse is clearly apparent with its active cleaving subunits in green color. The DNA components of the recombination complex are shown in blue and red backbone form. The tridimensional model is referenced as PDB 1NZB (Guo, Gopaul and van Duyne 1997). **(B)**Site-specific recombination model. Schematic representation of the consecutive reactions leading to the formation of recombinant DNA molecules by tyrosine recombinases. The color code is consistent with that of Panel A. The active tyrosine residues are shown in purple color.

The first full length tyrosine recombinase structure was obtained for the *E. coli* XerD resolvase and consisted of two domains separated by a disorganized linker (Subramanya *et al*. 1997). Subsequently, the resolution of the co-crystal structure of phage P1 Cre recombinase with its Lox site led to a structural model of the site-specific recombination reaction catalyzed by tyrosine recombinases (Guo, Gopaul and van Duyne 1997) (Fig. 8A). Both XerD and Cre crystal structures harbored an unfolded linker that separates the N-terminal domain from the C-terminal catalytic domain. The structure of the yeast 2µ plasmid Flp recombinase tetramer bound to an Holliday junction later revealed that the helix containing the nucleophilic tyrosine is swapped to cut the DNA in*trans* (Chen *et al*. 2000). When DNA cleavage occurs in*trans*, an integrase monomer activates the sessile phosphodiester bond while the adjacent monomer supplies the catalytic tyrosine. On the contrary, in the XerD and Cre structures, DNA cutting occurs in*cis* meaning that the same integrase monomer supplies the entire active site, as for the majority of bacterial integrases (Jayaram *et al*. 2015). The crystal structures of phage λ Int with its DNA substrates showed the simultaneous binding of two separate protein domains to DNA and suggested that the additional arm binding shifted the reaction equilibrium toward recombinant products (Biswas *et al*. 2005).

The only structural studies devoted to archaeal tyrosine recombinases concerned the integrase from the spindle-shaped virus SSV1 of *Sulfolobus shibatae* (suicidal integrase class) (Zhan *et al*. 2012), the resolvases PaXerA from *Pyrococcus abyssi* (Serre *et al*. 2013) and XerA from *Thermoplasma acidophilum* (TaXerA) (Jo *et al*. 2016). All three structures unsurprisingly revealed that archaeal tyrosine recombinases present a catalytic fold similar to bacterial and eukaryotic integrases (Eilers, Young and Lawrence 2012; Serre *et al*. 2013; Jo *et al*. 2017). Moreover, archaeal PaXerA and TaXerA proteins display the canonical structure of tyrosine recombinases comprising two domains surrounding the DNA substrate in a C-shape conformation (Serre *et al*. 2013) (Fig. 8A). The active sites of PaXerA and TaXerA assemble in*cis* (Jo *et al*. 2016) whereas IntSSV1 and *Thermococcus nautili* IntpTN3 catalyze DNA cleavage in*trans* (Letzelter, Duguet and Serre 2004; Eilers, Young and Lawrence 2012; Cossu *et al*. 2017). At this stage, only the structures of the catalytic C-terminal domain of archaeal recombinases have been resolved. The future resolution of the complete structure of archaeal tyrosine recombinases especially while bound to their DNA substrate might highlight further archaeal peculiarities.

## THE MECHANISM OF SITE-SPECIFIC RECOMBINATION

### A common catalytic mechanism for different reaction directionalities

The standard site-specific recombination reaction requires a tetramer of recombinases and a pair of identical DNA sequences specific to the enzyme involved (Grindley, Whiteson and Rice 2006) (Fig. 8B). The identity constraint can however be relaxed for one of the two sequences (Rajeev, Malanowska and Gardner 2009). The first stage of the reaction corresponds to the recruitment of the integrases to the specific site and to their tetramerization, resulting in the formation of a synaptic complex. The recombinases then catalyze two coordinated staggered single strand DNA cuts through the formation of a transient 3′-phosphodiester covalent bond between the active tyrosine and DNA (Grindley, Whiteson and Rice 2006). Then, strand exchange occurs between the two DNA segments and a nucleophilic attack by the 5′-terminal hydroxyl group of the invading strand resolves the covalent complex. The process is repeated leading to a total of four recombination reactions.

**Figure 9.**
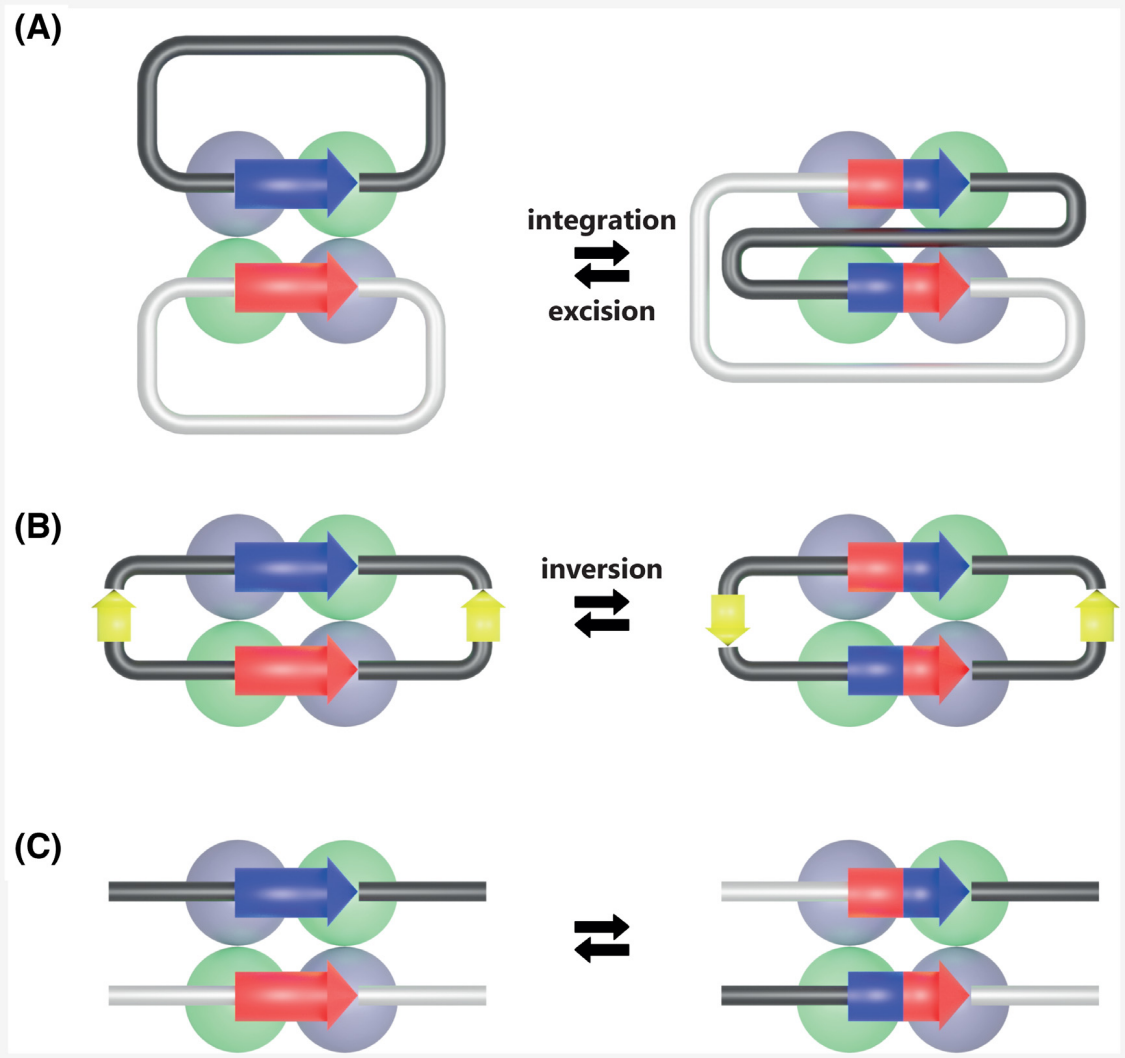
The different outcomes of site-specific recombination. The red and blue rectangles correspond to the specific recombination sites. With circular DNA molecules as substrates, the recombination outcome can be **(A)** integration and excision or **(B)**inversion, depending on the relative position of the two specific sites. **(C)**Recombination between two linear DNA molecules results in two chimeric linear DNA molecules.

Depending on the topology linkage of the two DNA sequences, the outcome of the recombination varies (Fig. 9A). Recombination between two sites carried by two independent circular molecules results in their integration. The newly formed chimeric circular molecule harbors the specific site in two copies in direct orientation. The inverse recombination reaction between these two sites produces an excision and the two initial circular molecules are restored. Recombination between two sites carried in opposite orientations by a single circular molecule produces an inversion (Fig. 9B). Integration corresponds to an intermolecular reaction while excision and inversions are intramolecular reactions. Site-specific recombinases can also catalyze recombination between two linear DNA molecules resulting in two chimeric linear DNA molecules (Fig. 9C).

### Helper proteins and recombination directionality factors

The symmetry of the synaptic complex illustrates the reversible nature of site-specific recombination reactions (Fig. 8B). In order to control the directionality of the reaction, small accessory proteins are often involved in stimulating one reaction while suppressing the other. These proteins are referred as recombination directionality factors or RDFs (Lewis and Hatfull 2001). The best-studied RDF is the phage λ-encoded Xis protein that is necessary for excisive recombination and inhibits integrative recombination (Abremski and Gottesman 1982). Phage λ excision is also favored by the host-encoded Fis protein (Papagiannis *et al*. 2007). The bacterial helper protein IHF contributes to both λ integration and excision reactions although integration requires more IHF than does excision for optimal reaction (Bushman *et al*. 1985). The completion of the resolution reaction of dimeric bacterial chromosomes by the XerC/D recombinases required the activity of the cell division protein FtsK (Barre *et al*. 2000).

Unessential archaeal cofactors were identified *in vivo* concurring in IntSNJ2 activity in *Natrinema*sp. J7-1 (Wang *et al*. 2018). The gene orf1 coding for the integrase is transcribed in an operon with two the other genes orf2 and orf3. Orf2 and orf3 code for small proteins (111 and 140 residues, respectively) containing a coiled coil motif that could mediate protein-protein interactions and a MarR-like DNA binding domain, respectively. The presence of one or both proteins increased IntSNJ2 *in vivo* integration activity by 30% (Wang *et al*. 2018). For the inversion reaction, the recombination efficiency was increased 70-fold in the presence of a single protein and 180-fold in the presence of both. They cooperatively activated IntSNJ2 recombination activity through an undetermined mechanism. Nevertheless, IntSNJ2 is active in their absence and the operons of many SNJ2-like viruses do not encode these cofactors (Wang *et al*. 2018).

### Site-specific recombination activity of unescorted archaeal integrases

**Figure 10.**
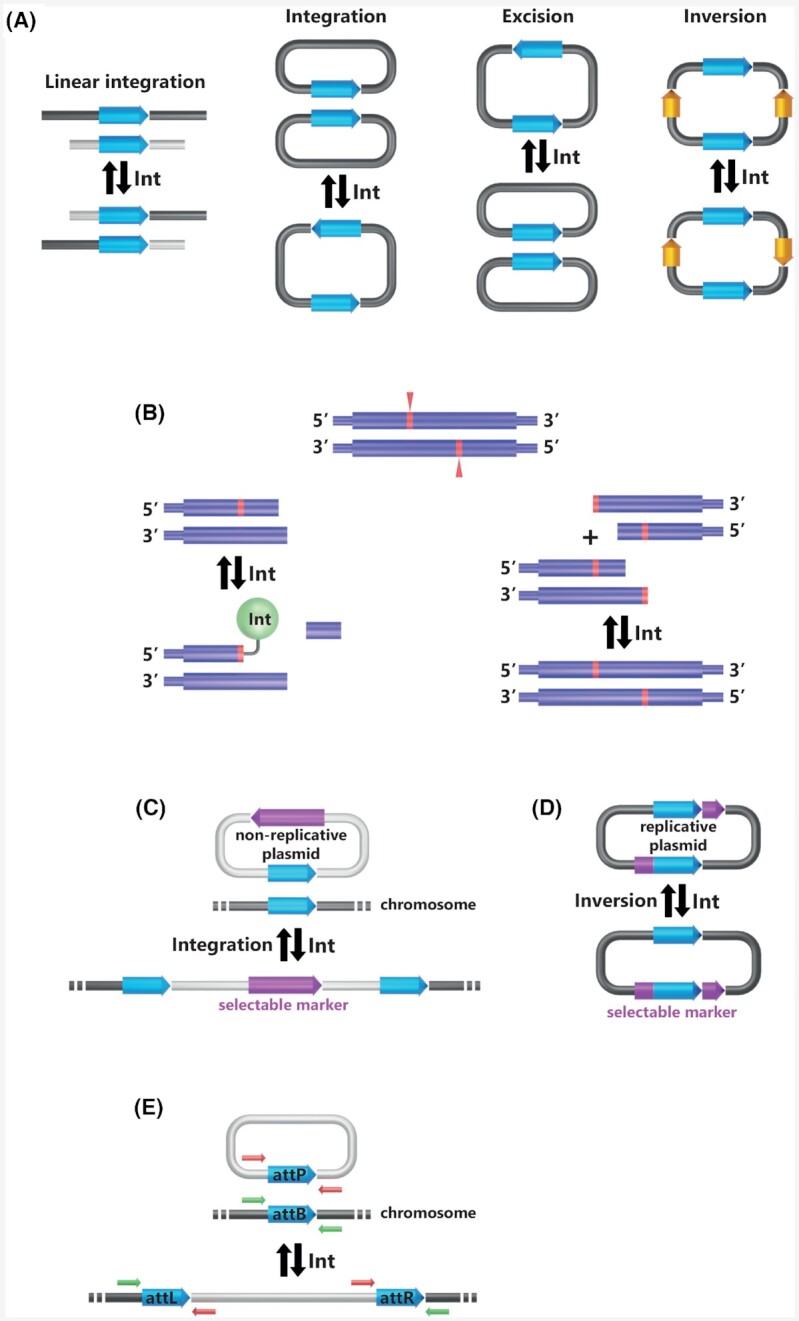
Assays to detect integrase activity *in vitro* and *in vivo*.**(A)** Different substrates harboring specific sites (light blue arrows) are incubated with the integrases *in vitro* and the products were monitored (Cortez *et al*. 2010; Zhan, Zhou and Huang 2015; Cossu *et al*. 2017; Jo *et al*. 2017; Badel *et al*. 2020). **(B)** The half-site strand transfer assay was first implemented *in vitro* by Serre *et al*. (2013). It allows the verification of the strand cleavage site (in red). The incubation of the integrase with half of the specific site results in a covalent DNA–protein complex that can be detected (left). The incubation of the integrase with two separated halves of the specific site results in the reconstruction of the entire specific site only if the substrate halves were designed accordingly to the cleavage site (right). **(C)** A non-replicative plasmid harboring the specific site and a selectable marker is introduced in a suitable host cell. Upon selection, only the cells where the integrase catalyzes plasmid integration can grow (Wang *et al*. 2018). **(D)** A replicative plasmid harboring two specific sites and a split selectable marker is introduced into the appropriate cell. The selectable marker is reconstituted only if the integrase catalyzes recombination between the two specific sites. The cell can then grow upon selection (Wang *et al*. 2018). **(E)** Different arrangements of the specific site result from integration or excision. They can be detected by polymerase chain reaction with different pairs of four primers (red and green arrows) (Li *et al*. 2016; Cossu *et al*. 2017; Wang *et al*. 2018).

The aforementioned molecular model can to all account be extended from bacterial and eukaryotic to archaeal tyrosine recombinases. In archaeal cells, site-specific recombination substrates are circular molecules. Several archaeal tyrosine recombinases were proven to be active *in vitro* and *in vivo* through various activity assays (Table 1; Fig. 10A). The first archaeal recombinase whose activity was tested *in vitro* is the *Sulfolobus* spindle-shape virus 1 integrase IntSSV1. The recruitment to a specific site and tetramerization was found to be rate limiting for IntSSV1 (Serre *et al*. 2002). Its recombinase activity observed *in vitro* by Muskhelishvili *et al*. (Muskhelishvili, Palm and Zillig 1993) could not be reproduced except for the first step of the recombination reaction, i.e. strand cleavage (Muskhelishvili, Palm and Zillig 1993; Serre *et al*. 2002; Letzelter, Duguet and Serre 2004; Zhan, Zhou and Huang 2015) (Fig. 10B). More recently, the tyrosine recombinases PaXerA from *Pyrococcus abyssi* and TaXerA from *Thermoplasma acidophilus*, typically resolving chromosome dimers *in vivo* were shown to catalyze integration reactions *in vitro* making of them *bona fide* integrases (Cortez *et al*. 2010; Serre *et al*. 2013; Jo *et al*. 2017). The two suicidal *Thermococcales* integrases IntpTN3 from plasmid pTN3 and IntpT26-2 from plasmid pT26-2, were also shown to catalyze site-specific recombination on circular substrates *in vitro*. (Cossu *et al*. 2017; Badel *et al*. 2020). All tested arrangements of specific sites allowed recombination in the absence of any additional cofactor. This suggests that, contrary to most bacterial integrases (Landy 2015), archaeal integrases do not require any RDF for efficient *in vitro* recombination. The integrases IntpTN3, IntSSV2, PaXerA and TaXerA could also catalyze site-specific recombination on linear substrates *in vitro* (Cortez *et al*. 2010; Serre *et al*. 2013; Landy 2015; Zhan, Zhou and Huang 2015; Cossu *et al*. 2017; Jo *et al*. 2017). Linear substrates are not their natural substrates but are useful to characterize some aspects of the integrase activity such as the strand cleavage site (Serre *et al*. 2013) (Fig. 10B). Additionally, three integrases (IntPYG1, IntpTN3, IntSNJ2) were shown to catalyze site-specific recombination *in vivo* (Li *et al*. 2016; Cossu *et al*. 2017; Wang *et al*. 2018)(Fig. 10C–E). Overall, the activity of several archaeal integrases from several families was characterized whose most remarkable aspect is the recurrent absence of necessary cofactor for catalysis, in stark contrast with most bacterial model integrases.

**Table 1.** Published archaeal tyrosine recombinase groups.

Representative integrase	Integrase type	Host order	Activity demonstrated	Biochemical analysis	Structure resolution	Reference
XerA	Classical	All chromosomally encoded	Yes	Yes	Yes	(Cortez *et al*. 2010; Jo *et al*. 2017)
pNOB8 integrase	Classical	*Sulfolobales*	No	No	No	(She, Brugger and Chen 2002)
PYG1 integrase	Classical	*Thermococcales*	Yes	No	No	(Li *et al*. 2016)
SNJ2 integrase	Classical	*Halobacteriales*	Yes	No	No	(Wang *et al*. 2018)
SSV1 integrase	Suicidal	*Sulfolobales* (*Desulfurococcales*)	Yes	Yes	Yes	(Serre *et al*. 2002; Zhan, Zhou and Huang 2015)
pTN3 integrase	Suicidal	*Thermococcales*	Yes	Yes	No	(Cossu *et al*. 2017)
pT26-2 integrase	Suicidal	*Thermococcales*, *Archaeoglobales* (*Methanosarcinales*)	Yes	Yes	No	(Badel *et al*. 2020)

### DNA relaxation activity of archaeal integrases

Despite the very limited overall sequence conservation observed in our network analysis, eukaryotic and bacterial tyrosine recombinases and topoisomerases IB share a common catalytic core that could have originated from an ancestral strand transferase (Cheng *et al*. 1998; Yang 2010). This relationship explains why tyrosine recombinases can often catalyze DNA relaxation (Abremski *et al*. 1986; Landy 2015). The archaeal recombinases IntpT26-2, IntSSV1 and TaXerA also presented non-specific DNA relaxation activities (Letzelter, Duguet and Serre 2004; Jo *et al*. 2017; Badel *et al*. 2020). This property underlines a similar relationship between archaeal tyrosine recombinases and topoisomerases IB. It is to be noted however that topoisomerases cleave and then join the same 5′ and 3′ termini, whereas site-specific recombinase transfer a 5′ hydroxyl to a new 3′ phosphate partner from a different strand.

## ARCHAEAL DNA RECOMBINATION TARGETS

### Attachment site characteristics

**Figure 11.**
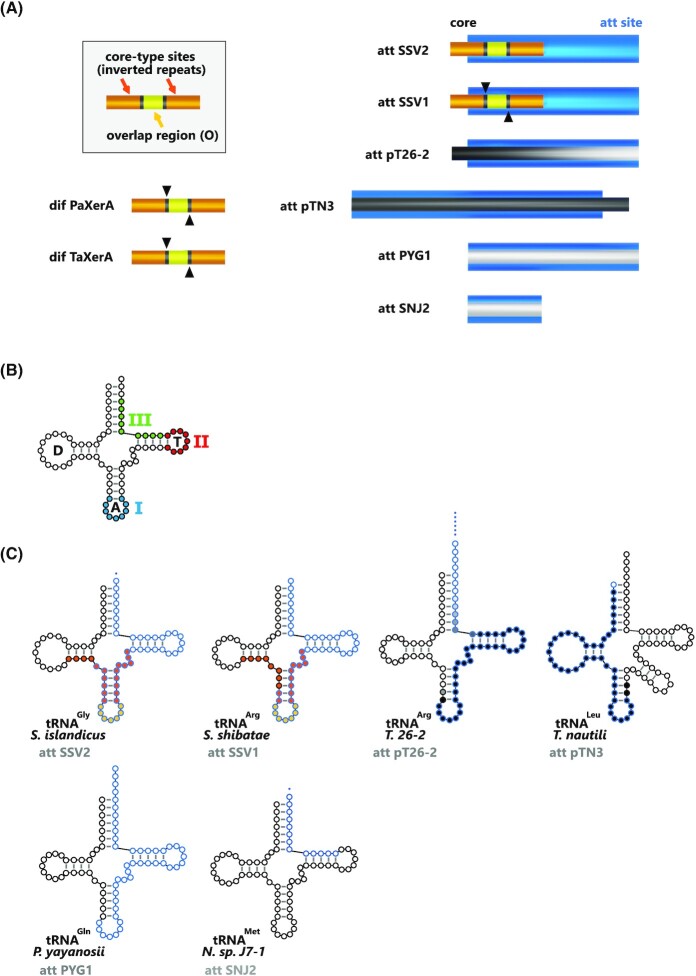
Archaeal tyrosine recombinase recombination sites.**(A)** Recombination sites are sketched for the characterized archaeal tyrosine recombinases. The orange and yellow boxes correspond to core-type sites as defined for bacteriophage λ (Landy 2015). The blue box corresponds to the att site. The black sequences are necessary and sufficient for recombination *in vitro* (Cossu *et al*. 2017; Badel *et al*. 2020). The black arrows indicate the cleavage site when experimentally determined (Serre *et al*. 2002, 2013; Jo *et al*. 2017). **(B)** General organization of a tRNA indicating the location of the T, A and D loops and the three preferred integration locations for bacterial integrases (I, II and III) (Williams 2002). **(C)** Att sites often correspond to tRNA sequences. The leaf-like structure of the targeted tRNA is indicated with att site nucleotides circled in blue.

The attachment sites define the DNA segments containing the points of strand exchange and the binding site for site-specific recombinases. These enzymes catalyze the recombination between the attP (attachment phage) site on the episomal MGE and attB (attachment bacteria) on the chromosome (Landy 2015) (Fig. 2). This reaction generates the two hybrid attL (attachment Left) and attR (attachment Right) sites bordering the integrated MGE. In the canonical model derived from the structure of the Cre/LoxP synapse, the four att sites are strictly identical and correspond to the recombinase specific site (Fig. 8). The lambdoid phages constitute a notable exception to this rule: their ∼240 bp attP site carries multiple binding sites for Int, Xis and IHF (Hsu, Ross and Landy 1980) whereas the ∼20 bp attB carries only two Int binding sites (Mizuuchi and Mizuuchi 1980). These attB and attP share an identical DNA stretch of 15 bp called the core containing the two points of strand exchange. The 7bp interval between these exchanges on the two strands is the overlap region (Craig and Nash 1983) (Fig. 11A).

Archaeal attachment sites have been defined by the extent of exact DNA sequence shared by MGEs and their host chromosome. They usually extend over 40 to 50 bp for suicidal integrases (She, Brugger and Chen 2002; Cossu *et al*. 2017), 40 to 50 bp for *Sulfolobales* pNOB8 integrases (She, Brugger and Chen 2002; Erauso *et al*. 2006) and 50 to 60 bp for Methanococcales integrases (Badel *et al*. 2020). They can be as short as 8 bp for *Thaumarchaeota* integrated elements (Krupovic *et al*. 2019), 11 bp for the *Methanobacteriales* Msmi-Pro1 integrated virus (Krupovic, Forterre and Bamford 2010) or 13 bp for the *Halovivax* SNJ2 integrated virus (Liu *et al*. 2015) or they can be longer than 100 bp for some *Thermococcales* elements (102 bp for PkuNCB100_IP1 and 243 bp for TIRI33c_IE1) (Badel *et al*. 2019, 2020). Interestingly, the large att site of these two *Thermococcales* MGEs encompassed the shorter att site of the closely related integrases from the PHV1 and TGV1 viruses, respectively (Badel *et al*. 2020).

Several studies aimed at characterizing experimentally the extent of archaeal att sequences required for efficient integration and postulated the existence of a minimal and sufficient recombination site. It appears however that att sites defined by a strict sequence conservation between MGE and host chromosomes are inoperative for recombination. For IntSSV1 and IntSSV2, two sequences were suggested to be sufficient for recombination *in vitro*: the *stricto sensu* att site or the inverted-repeats separated by an overlap region, reminiscent of the phage λ core (Muskhelishvili, Palm and Zillig 1993; Serre *et al*. 2002; Zhan, Zhou and Huang 2015) (Fig. 11A). These two sequences did not completely overlap and the sequence at the intersection was not assayed for recombination. The minimal site remained therefore undefined. For IntSSV1, the attB strand exchanges were observed in a tRNA gene with the 5′ cuts bordering an overlap region that corresponded to the tRNA anticodon loop as for classical bacterial tyrosine recombinases (Serre *et al*. 2002; Grindley, Whiteson and Rice 2006). However, the *in vivo* observation of non-specific integration events suggested that the strand cleavage position could vary (Wiedenheft *et al*. 2004). For both IntpTN3 and IntpT26-2, the identical DNA stretch shared by the attL and attR sites is not sufficient for recombination *in vitro* (Cossu *et al*. 2017; Badel *et al*. 2019, 2020). Additional nucleotides are required in order to encompass the anticodon loop and the proximal stem extremity (Fig. 11A–C). It could be extrapolated that the cleavage sites for IntpTN3 and IntpT26-2 border the anticodon loop as reported for IntSSV1 or the D and T loops, respectively (Fig. 11B). A cleavage site at the extremities of a tRNA loop can also be considered for IntPYG1 but not for IntSNJ2 whose att site is very short (Fig. 11A–C). It would be interesting to determine whether the 14 nt long and stem loop-free att site from IntSNJ2 is sufficient for recombination. Finally, for IntpT26-2 recombination, the nucleotides of the acceptor stem are not necessary but their presence significantly increases recombination efficiency. The recombination site does not seem to be a precisely defined and finite sequence but rather a stretch of nucleotides that favor recombination. The effective recombination site of IntpT26-2 is not located at the center of the att site but shifted toward its 5′ end, similarly to IntSSV1 (Serre *et al*. 2002; Zhan, Zhou and Huang 2015) and numerous bacterial integrases (Campbell 1992). Overall, archaeal attachment sites are reminiscent of their bacterial counterparts but present the peculiarity to require additional nucleotides outside the conserved sequence between attB and attP.

If most integrases display a marked preference for a particular specific site on the host chromosome, they can also target slightly different sequences albeit with a reduced efficiency. Phage λ Int could recognize many such sites whose sequence deviated from the original att while retaining structural features such as the twist and roll angle between adjacent base pairs (Nussinov and Weisberg 1986). The presence of secondary attachment sites could play a determinant role in the specify switch and evolution of integrases (Rutkai *et al*. 2006). The existence of secondary attachment sites was investigated for integrases of archaeal fuselloviruses. The SSV2 virus was only found integrated in its cognate attB site and not in any other slightly divergent site (Contursi *et al*. 2006). The recombination site specificity was found to be more relaxed for the SSV1 virus that, in absence of its cognate attB site, could integrate in a sequence differing in two nucleotides (Schleper, Kubo and Zillig 1992; She *et al*. 2001a; Contursi *et al*. 2006).

### Most integration sites reside in tRNA genes

The abundance of attachment sites located within tRNA genes has been extensively documented for bacterial integrases (Williams 2002). Due to the conservativeness of site-specific recombination, an intact tRNA gene sequence is reconstituted after MGE integration. With very few exceptions, the recombinant bacterial tRNA gene is expressed from its original promoter (Williams 2002). The preference for these targets is due in part to the remarkable structural similarities between tRNA genes features and the canonical attachment site defined by phage λ attB. DNA segments corresponding to the usually 7 bp-long anticodon loop and flanking palindromic stem match remarkably the consensus 7 bp overlap region and core site organization (Campbell 2003). This similarity suggests that tRNA genes are common integration sites because they were the target of a primordial tyrosine recombinase (Reiter, Palm and Yeats 1989; Campbell 1992). Additional reasons were invoked for the selection of tRNA genes as preferential attachment targets. First, all tRNA genes harbor several characteristic regions of dyad symmetry that could serve as binding sites for recombinases (Reiter, Palm and Yeats 1989). Second, tRNA genes are very stable through time (Williams 2002), it is therefore possible that ancestral tyrosine recombinases targeting other sequences disappeared when their target sequence changed. Finally, tRNA genes belong to a multigene family offering a multitude of potential target sites with only limited nucleotide changes (Winckler, Szafranski and Glockner 2005).

Archaeal tRNA genes constitute preferential integration targets as well (She, Brugger and Chen 2002; Cossu *et al*. 2017; Wang *et al*. 2018; Badel *et al*. 2019,2020; Krupovic *et al*. 2019). Recently, a systematic survey of all integrated MGE in thaumarchaeal genomes showed that more than half of the attB sites were located in tRNA genes (Krupovic *et al*. 2019). Additionally, half of the tRNA genes present in *Thaumarchaeota* were used as integration site at least once, including tRNA genes with introns (Krupovic *et al*. 2019). The evolutionary stability of tRNA genes sequences is also exploited by archaeal integrases. This sequence conservation allowed the SSV2 virus to integrate in multiple genomes such as those of *Sulfolobus islandicus* and *S. solfataricus* (Contursi *et al*. 2006). Similarly, the closely related integrases form plasmid pXZ1 and SSVA fusellovirus targeted two separate tRNA^Glu^ genes differing by a single nucleotide (Peng 2008).

In a more detailed approach, it appeared that three different regions of the tRNA gene can be used for bacterial integration (Williams 2002). Two of these regions, the anticodon stem loop and the T stem loop contain a dyad symmetry whereas the third region, located at the 3′ end, has no symmetry (Fig. 11B). While archaeal integrases have been found to target the same three regions, a preference emerged for the 3′ end of tRNA genes with various 5′ limits (Serre *et al*. 2002; Badel *et al*. 2019, 2020). The archaeal att site could be somewhat larger and overlapped both the anticodon loop and the T stem loops as for SSV2 integration (Contursi *et al*. 2006) or could be as short as the amino acid attachment site as for SNJ2 integration (Liu *et al*. 2015). The pTN3-like integrases were found to be unique in archaea in that their attB site corresponds to the 5′ half of the tRNA gene (Cossu *et al*. 2017). As it is for all integration events, pTN3-like integrases restore an uninterrupted copy of the original tRNA gene. A notable difference resides in the fact that these recombinant tRNA genes are expressed from the integrase promoter (Krupovic and Bamford 2008; Cossu *et al*. 2017). A similar situation has been seldomly encountered in bacteria (Williams 2002). In rare documented event, the non-specific integration of virus SSVK1 into a tRNA^Glu^ gene sporadically generated a tRNA^Asp^ gene (Wiedenheft *et al*. 2004).

**Figure 12.**
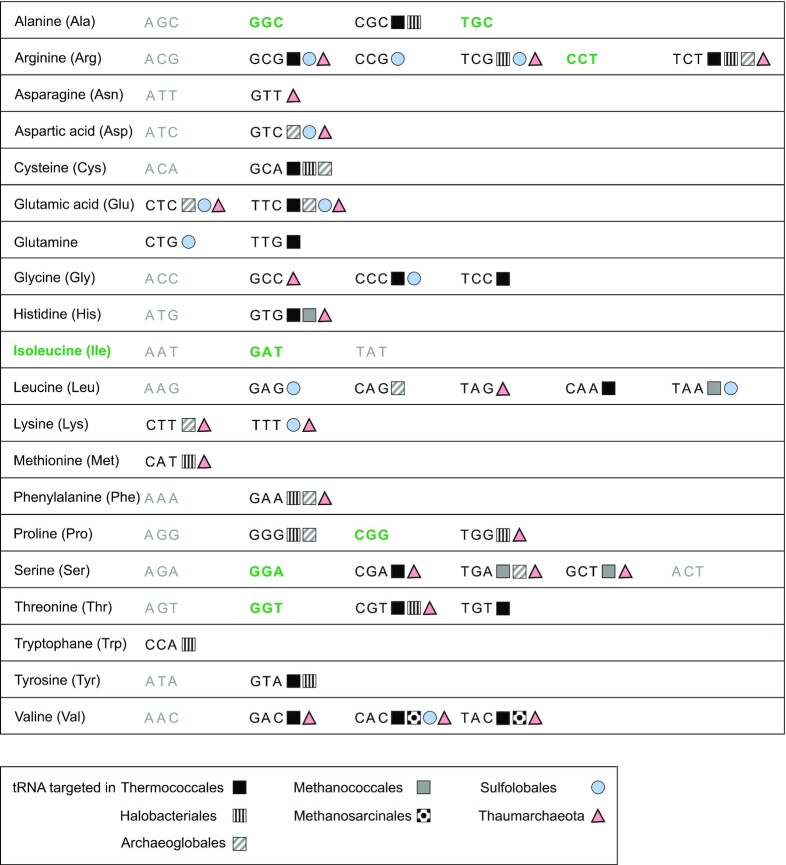
Wide tRNA genes targeting by archaeal tyrosine recombinases. All tRNA anticodon combinations are listed along with their corresponding amino acids. Anticodons that are not found in archaeal tRNAs are indicated in light gray. An array of symbols indicates the utilization of a tRNA gene with the specified anticodon as attB site for *Thermococcales* (Li *et al*. 2016; Cossu *et al*. 2017; Badel *et al*. 2019, 2020), *Halobacteriales* (Krupovic, Forterre and Bamford 2010; Liu *et al*. 2015), *Archaeoglobales* (Badel *et al*. 2020), *Methanococcales* (Krupovic, Forterre and Bamford 2010; Badel *et al*. 2019), *Methanosarcinales* (Badel *et al*. 2020), *Sulfolobales* (She, Brugger and Chen 2002; Wang *et al*. 2007; Peng 2008; Redder *et al*. 2009) and *Thaumarchaeota* (Krupovic *et al*. 2019). Anticodons corresponding to untargeted tRNA genes are highlighted in bold green.

We inventoried the tRNA genes used as integration sites in archaea and observed marked differences in the targeting frequencies of the various tRNA genes (Fig. 12). Out of the collection of 44 tRNA genes, only 7 were never targeted suggesting that their use as integration site would be deleterious. On the other hand, tRNA^GluTTG^, tRNA^ArgTCT^ and tRNA^ValCAC^ were used more frequently than the others. By comparing preferred codon usage and integration frequency in all sequenced *Thermococcales*, it appeared that targeting occurs preferentially in genes encoding tRNAs that read rare codons (our unpublished observation). This result is consistent with the observation that the tRNA genes most frequently used as integration targets by *E. coli* phages were the least expressed and corresponded to the rarer codons (Bobay, Rocha and Touchon 2013).

In general, archaeal integrases select their tRNA gene targets following the same rules as bacterial integrases with some notable exceptions. Archaeal attB sites can be somewhat larger and extend over the tRNA D-loop and even outside the tRNA gene sequence.

### Integration in other intragenic sequences and intergenic regions

Genes encoding tRNAs are not the sole targets of archaeal integrases. Several archaeal attB sites were reported in intergenic sequences or within protein coding genes (Luo *et al*. 2001; Krupovic, Forterre and Bamford 2010; Krupovic *et al*. 2019). The att sites of prophage ΨM100 of *Methanothermobacter wolfeii*, correspond to an intergenic region and prophage integration has no effect on adjacent gene transcription (Luo *et al*. 2001). It is noteworthy that prophage ΨM100 is a *Siphoviridae* like phage λ (Brussow and Desiere 2001) and targets an AT-rich att site in an intergenic region similarly to phage λ (Campbell 1992). Att sites are also found in coding regions (Krupovic, Forterre and Bamford 2010). When the function of the targeted gene is known, it can be as diverse as a gene coding for 3-hydroxy-3-methylglutaryl-coenzyme A reductase for the *Halorubrum*sp. virus BJ1 (Krupovic, Forterre and Bamford 2010), for heavy metal cation efflux system for the *Methanobrevibacter smithii* provirus Msmi-Pro1 (Krupovic, Forterre and Bamford 2010) or for AsnC family transcriptional regulator gene for the integrated element NitGar-E6 of Candidatus *Nitrososphaera gargensis* Ga9.2 (Wang *et al*. 2018). Recombination was not tested *in vivo* or *in vitro* with these inter- and intra-genic regions. It would be interesting to determine their efficiency for recombination and which positions are essential. Especially, since the presence of dyad symmetry in their DNA substrates is crucial for the activity of most tyrosine recombinases analyzed to date, we wonder whether those genes present such features.

### Dimer resolution at dif sites

The integration of MGE sequences into their host genome does not constitute the sole function of site-specific recombinases. As for bacteria, archaeal chromosomally-encoded Xer tyrosine recombinases exert an essential role in genome maintenance and integrity. The sites of Xer recombinases are named dif and are present in single copy on circular chromosomes of archaea and bacteria (Castillo, Benmohamed and Szatmari 2017; Cossu *et al*. 2017). The Xer/dif system improves cell viability by resolving concatenated chromosomes occurring by homologous recombination during DNA replication. When a chromosome dimer is formed, the dif sequence appears duplicated and the dimer is resolved through site-specific recombination between the two dif sites. In bacteria and archaea, the dif sequence is composed of two 11-nt inverted repeats separated by a 6-nt spacer (Cortez *et al*. 2010; Cossu *et al*. 2017; Jo *et al*. 2017). The extremities of the spacer correspond to the position of the tyrosine-catalyzed cleavage (Serre *et al*. 2013; Castillo, Benmohamed and Szatmari 2017). The dif sequence is variable to a certain extent except for some conserved positions (Cortez *et al*. 2010). It seems that both the stem loop structure and the nucleotide sequence are important for the functionality of dif sites. Interestingly, PaXerA can bind its dif substrate with a high affinity and stem-looped structures of unrelated att sites with a lower affinity (Cortez *et al*. 2010). In the bacterial model of dif recombination, single XerC and XerD monomers each bind to an inverted repeat of the dif site (Castillo, Benmohamed and Szatmari 2017). Similarly, TaXerA and PaXerA can bind each dif inverted repeats (Serre *et al*. 2013; Jo *et al*. 2017). The activity of TaXerA was assayed on a series of dif site variants mutated in the inverted repeats (Jo *et al*. 2017). All variants allowed recombination even if to a lesser extent than the wild-type dif site. Depending on the variant, the reduced activity was due to reduced binding affinity or reduced strand exchange (Jo *et al*. 2017) indicating that key dif positions are involved either in sequence recognition or in the strand-transfer reaction. Due to the deleterious effect of chromosome dimers at cell division, it is vital that the equilibrium of the reversible Xer/dif reaction be displaced toward resolution. In bacteria, this function is officiated by the cell division protein FtsZ (Barre *et al*. 2000) whereas no equivalent system has been identified in archaea so far.

## ECOLOGY OF ARCHAEAL INTEGRATION

Integrases are responsible for integration into and excision from a chromosome, the central switches in mobile genetic elements life history. As such, integrase catalytic activities should be considered in the light of the ecological consequences of the mobile genetic element lifestyle. In this section, we present the current knowledge about the host specificity of integrases and the type of mobile genetic elements that encode them. We also discuss the advantages of encoding an integrase for mobile genetic elements and the control of integrase directionally and temporality.

### Integrase host specificity

Tyrosine integrases are detected in many archaeal phyla but closely related integrases are mostly found in a narrower range of organisms indicating a certain degree of host specificity (Fig. 6 and Table 1). In *Methanococcales* and *Thermococcales* that were thoroughly investigated, it was found that plasmids related to pT26-2 harbor two distinct integrase families (Badel *et al*. 2019). Furthermore, it was observed that similar integrases from pleolipoviruses were present in the chromosomes of 10 different *Halobacteriaceae* genera from various geographical locations (Liu *et al*. 2015). Related integrases are not restricted to a local area but are found all around the globe where their host is present.

### Mobile element recruitment

In the archaeal domain, integrases are carried by a wide variety of MGEs: conjugative plasmids (She, Brugger and Chen 2002), cryptic plasmids (Erdmann *et al*. 2017; Badel *et al*. 2019), viruses from several viral families such as *Myoviridae* (Klein *et al*. 2002; Tang *et al*. 2002), *Pleolipoviridae* (Atanasova *et al*. 2018a), *Fuselloviridae* (Goodman and Stedman 2018) and unidentified MGEs (Li *et al*. 2016). The *Thermococcales* pT26-2 family of integrases is present in plasmids from the pT26-2 family, in plasmid from the pAMT11 family, in Fusellovirus and in unidentified MGEs (Badel *et al*. 2020). Similarly, two very similar integrases were identified in *Sulfolobus solfataricus*, on a plasmid and a virus (86% nucleotide similarity, 94% amino acid similarity) (Peng 2008) indicating that integrases from the same family can be recruited by several mobile elements. Additionally, some MGE families include integrative and non-integrative members as the *Thermococcales* pAMT11 family (Argos *et al*. 1986) or the haloarchaeal pleolipoviruses (Roine *et al*. 2010; Liu *et al*. 2015). Strikingly, the sequences of the two pleolipoviruses HHPV3 and HHPV4 are very syntenic and similar except for a 3 kb HHPV4-specific region carrying an integrase gene (Atanasova *et al*. 2018a). This situation could result either from integrase acquisition or loss for some MGE members. For suicidal integrases, the att site is included within the integrase gene resulting in a compact module that could favor exchange between mobile elements (Ausubel 1974). The pING1 plasmid was identified as encoding an integrase exhibiting all the conserved residues of its family of pNOB8-like integrases but no attP site could be determined (Erauso *et al*. 2006). It is possible that in that case, attP site loss would lead to integrase gene degeneration and/or loss. Finally, even when the integration module is conserved in a plasmid family, its evolutionary history can be complex. This is observed in all the conjugative *Sulfolobales* plasmids that exhibit conserved conjugation and integration modules. However, the phylogenetic trees of the two modules are not congruent suggesting intrafamily module exchanges (Erauso *et al*. 2006). On the whole, the frequent integrase exchange between mobile elements is featured in a network of all archaeal viruses where some integrases represent connector genes between virus clades (Iranzo *et al*. 2016). However, in the network, other integrases represent a signature gene of a clade evidencing their favored residence in those particular MGEs. Some archaeal integrases seem ‘settled’ (Iranzo *et al*. 2016) whereas the majority is frequently exchanged, gained or lost between MGEs.

### Integration is a major lifestyle for archaeal mobile elements

The primary function of tyrosine integrases is to catalyze the integration of the MGE that encode them into the host chromosome or the reverse reaction of excision (Fig. 2). Such integrase-encoding MGEs are widely present in archaeal genomes (Soler *et al*. 2010; Gaudin *et al*. 2014; Wang *et al*. 2018; Krupovic *et al*. 2019). In *Thermococcales*, it was shown that >30% of the published genomes contain an integrated element encoding an integrase of the pT26-2 family (Iranzo *et al*. 2016). The proportion of genomes presenting any integrase-encoding MGE is most probably higher than that. In the phylum *Thaumarchaea*, integrated MGEs were systematically detected and found in 20 out of 21 analyzed genomes (Krupovic *et al*. 2019). In halophilic archaea, SNJ2-like integrases from integrated MGEs form a large, well-supported clade with the MGE-encoded hyperthermophilic integrases (Wang *et al*. 2018). In this systematic search, several integrated MGEs would not encode an integrase. This presumably results from the integrase gene loss after integration similarly to what was observed for integrated plasmids of *Methanococcales* (Badel *et al*. 2019). Several related or unrelated MGEs can be integrated in the same chromosome at different loci (Pauly *et al*. 2019; Badel *et al*. 2020) or integrated in tandem at the same locus (Krupovic, Forterre and Bamford 2010; Krupovic *et al*. 2019). No account for λ-type immunity system has been reported for archaeal MGEs therefore enabling co-infection or superinfection. Overall, integrated MGEs are widely present in archaeal genomes suggesting strong evolutionary advantages for integration in this domain (Fig. 6).

### Advantages of mobile element integration: why code for an integrase?

Advantages were uncovered for phage λ lysogenic state: integration increases long-term MGE maintenance (Echols 1972) and the integrated state cell provides a solution when chances of finding a new suitable host are low (Levin, Stewart and Chao 1977). The canonical integration model established for phage λ proposes that the integrative state would help the MGE to survive through adverse environmental conditions. During lysogeny, the MGE genome only exists in the integrated form and is silenced. When stressful conditions are encountered by the cell, the MGE excises and enters the lytic cycle and virions are released into the environment though cell lysis (Paul 2008; Gandon 2016). Depending on the environmental conditions, the MGE chooses to reproduce vertically (integration) or horizontally (infection) though highly controlled mechanisms. The same lifestyle was observed for *Acidianus* convivator bicaudavirus ATV (Prangishvili *et al*. 2006). Under optimal growth temperature conditions, it adopts a lysogenic lifestyle and integrates into the host chromosome. Inversely, under suboptimal growth temperature conditions, the virus adopts a lytic lifestyle resulting in host cell lysis.

For the archaeal fuselloviruses, which are the most studied archaeal MGEs encoding an integrase and exemplified by the model virus SSV1, the integration implications differ on several aspects from the lysis/lysogeny switch paradigm of phage λ (Prangishvili, Stedman and Zillig 2001). (i) SSV1 viral production is induced by a UV irradiation (Martin *et al*. 1984), mitomycin C treatment (Liu and Huang 2002) or by shaking the culture (Liu and Huang 2002) similarly to phage λ, but cells do not lyse massively after viral production and return to the lysogenic sate (Martin *et al*. 1984). Virus TPV1 replication is also induced by UV-treatment without any extensive cellular lysis (Gorlas *et al*. 2012). (ii) During the SSV1 integrative stage, a few circular copies of the viral genome remain in the cell (Yeats, McWilliam and Zillig 1982; Pauly *et al*. 2019). Similarly, a high copy number of TPV1 circular DNA is present in its host cells (Gorlas *et al*. 2012). (iii) During the SSV1 integrative stage, the majority of the viral ORFs are expressed, including the integrase gene and the structural proteins (Frols *et al*. 2007). It is not known whether the transcription template corresponds to the integrated or episomal copy of the viral genome. A transcriptional regulator was identified that is probably involved in lysogeny regulation (Fusco *et al*. 2015b) but it does not result in provirus silencing as it is the case for phage λ. Contrastingly, the SSV2 integrase is not basally expressed (Fusco *et al*. 2015b). (iv) For the virus SSV1, evidence point toward the replication of already present circular DNA independently of the integrated copy rather than an excision and subsequent replication of the circular DNA similarly to λ (Fusco *et al*. 2015b). Overall, and contrarily to the lambdoid paradigm, it seems that the integrase of lysogenic fuselloviruses is not involved in the regulation of virus replication and virion production. Nevertheless, most fuselloviruses encode a suicidal tyrosine integrase (Gorlas *et al*. 2012; Goodman and Stedman 2018) suggesting a probable evolutionary importance for virus survival. SSV1 viruses lacking the integrase gene were found to be outcompeted by wild-type viruses (Clore and Stedman 2007). However, mutant viruses were infectious and stably maintained in *Sulfolobus* and no clear benefit was associated with integrase activity. The exact evolutionary advantage of fusellovirus integrase still remains to be determined.

A number of archaeal plasmids were identified that are present in the cell both in the integrated and episomal states (Basta *et al*. 2009; Gaudin *et al*. 2014; Cossu *et al*. 2017). Contrarily to highly controlled lysis/lysogeny switch of temperate phages, archaeal plasmids such as pTN3 and pAH1 use a rudimentary safekeeping mechanism. Integration appears in this case as a simple and efficient solution to ensure the propagation of replicative plasmids when targeted by host defenses or other superinfecting MGEs. The initial isolate of *Thermococcus nautili* carried plasmid pTN3 in both replicative and integrated forms whereas the circular form was lots after successive subculturing (Cossu *et al*. 2017). This plasmid loss was caused presumably by the clustered regularly interspaced short palindromic repeats defense system (CRISPR-Cas9) (Oberto *et al*. 2014). Similarly, the *Acidianus hospitalis* pAH1 plasmid was evidenced to be stably maintained simultaneously in integrated and episomal states (Basta *et al*. 2009). When the host was co-infected with the virus AFV1, the episomal form disappeared rapidly while the integrated form persisted. These observations suggest that the integrated form can act as a safekeeping copy of the disappearing plasmid.

Integrating the host chromosome might force the cell into accepting the MGE and shutting down its defense systems. The targeting of integrated MGEs by the CRISPR system might induce an autoimmune response and death of the infected cell (Stern *et al*. 2010; Wimmer and Beisel 2019). It was postulated also that multiple integrations of related fuselloviruses and frequent recombinations among their highly similar genomes might provide a means to evade their hosts CRISPR system (Redder *et al*. 2009). Transcriptional activation of the CRISPR-Cas system was observed during SSV2 fusellovirus infections leading to a significant reduction in SSV2 copy number, its integration into the host chromosome and the deletion of several repeats-spacer units from the CRISPR array (Fusco *et al*. 2015b). As a result, all copies of the intact integrase gene were lost abolishing excision and effectively trapping the provirus in the chromosome. From a population genetics point of view, MGEs encoding suicidal integrases could be considered ‘kamikazes’ which role would be to defeat host defense mechanisms.

Integrated MGEs can provide functions that are beneficiary for the host (Schuch and Fischetti 2009; Wang *et al*. 2010) and therefore increase the probability of MGE retention. *Thermococcus kodakarensis* mutants lacking each of the four integrated TKV1 to TKV3 elements displayed impaired growth suggesting their importance for cellular metabolism at least in laboratory conditions (Tagashira *et al*. 2013). Similarly, the integrated element PYG1 was shown to increase its host resistance to temperature (Li *et al*. 2016).

### Integration/excision temporality control in archaeal mobile elements

The control of MGE integration and excision was thoroughly investigated for the bacterial lambdoid phages evidencing a complex regulatory genetic network (Oppenheim *et al*. 2005). Two levels of regulation were observed: (i) reaction temporality control and (ii) reaction directionality control (integration or excision). It is interesting to investigate whether integration and excision are also tightly regulated in archaea and if similar regulatory networks are implemented. In *Pyrococcus abyssi*, it was proposed that the integrase of the genomic island PYG1 can spontaneously catalyze excision since PYG1 does not carry an identified replication module and the element can be found in a circular state (Li *et al*. 2016). MGE excision seems in that case loosely controlled.

The first level of integration/excision temporal regulation consists in the regulation of integrase transcription. In some pNOB8-like integrases, the presence of a HTH domain was proposed to be involved in the transcriptional regulation of the integration/excision of the MGE (Erauso *et al*. 2006). For the *Sulfolobus* spindle-shaped viruses, transcription temporality was investigated by several studies. In SSV1, the integrase is under the control of an early promoter that allows a rapid expression after UV-induction (Frols *et al*. 2007) and the F55 repressor downregulates expression of the integrase operon in the absence of induction (Fusco *et al*. 2013, 2015b). Contrastingly, the integrase from virus SSV2 is expressed in the late infection phase consistently with the provirus detection >7 h after infection (Ren, She and Huang 2013). Moreover, SSV1 and SSV2 integrases are expressed from polycistronic operons while for other SSV viruses, the integrase is proposed to be translated from a monocistronic mRNA transcript (Goodman and Stedman 2018). The mechanisms of integrase expression regulation in the various SSV viruses appears to be diverse but still remains largely unexplored.

Some archaeal halophilic tailed viruses belong to the Caudovirales, which also include tailed bacteriophages (Sencilo *et al*. 2013; Krupovic *et al*. 2018). Among them, the archaeal Myovirus φCh1 can integrate into its host genome (Witte *et al*. 1997) and two potential tyrosine integrase sequences were identified (Klein *et al*. 2002). φCh1 regulatory network for the switch from the lysogenic to the lytic cycle was partially elucidated (Iro *et al*. 2007; Selb *et al*. 2017) and involved Rep, a repressor protein that functions convergently to phage λ cI repressor protein (Iro *et al*. 2007). During λ lysogeny, the specific binding of cI to its operator sites embedded in promoter sequences induces its own expression but represses the transcription of the lytic operons. A similar repressor protein is also present in the non-integrative myovirus φH1 (Ken and Hackett 1991; Stolt and Zillig 1992) suggesting that it might be implicated in the regulation of virion production rather than in excision control. Proteins similar to the repressor were also found in several integrase-encoding Pleolipoviruses (Chen *et al*. 2014; Liu *et al*. 2015; Atanasova *et al*. 2018a) suggesting that this mechanisms of lysis-lysogeny regulation is widely shared among halophilic viruses.

### Integration/excision directionality control in archaeal mobile elements

All the characterized archaeal integrases can catalyze both integration and excision reactions in the absence of any recombination directionality factor (RDF) in sharp contrast to the phage λ directionality regulation (Landy 2015). However, the activity of the halophilic integrase IntSNJ2 is modulated by two proteins Orf2 and Orf3, which increased *in vivo* integration efficiency (Wang *et al*. 2018). Orf1 to 3 are transcribed in an operon with two alternative transcription start sites. Using one or the other transcription site might constitute a control system for lysogeny.

In experimental setups with complete integrase proteins, characterized suicidal integrases catalyzed integration and excision alone (Cossu *et al*. 2017; Badel *et al*. 2020). However, in naturally occurring conditions, suicidal integrases are partitioned after integration. Excision would then require the activity of the split integrase that might be inactive. As a consequence, excision could not proceed after integration in the absence of some external factor (a complete integrase gene). This situation is similar to the directionality control by a RDF except that, for suicide integrases, the RDF is the complete integrase gene. In that sense, the suicidal integrase can be viewed as an ‘all in one integration module’ that include the integrase gene, the recombination site and the recombination directionality factor.

## INTEGRASE EVOLUTION AND SPECIFICITY SWITCH

### Tyrosine recombinases evolution

The first extensive tyrosine recombinase alignments revealed that the C-terminal portion carrying the catalytic domain is much more conserved than the N-terminal part responsible for site-specific recognition and protein multimerization (Esposito and Scocca 1997; Guo, Gopaul and van Duyne 1997). This variability reflects the divergence in target site sequence and the capacity of some phage integrases to bind to two distinct DNA segments (Moitoso de Vargas *et al*. 1988). The sequence divergence of tyrosine recombinases clearly illustrates the ancient origin of these proteins. The detailed evolutions mechanisms leading to such a diversity and particularly to the acquisition of different specificities are not fully understood. The naturally occurring change in specificity between related recombinases has been addressed for the integrases of lambdoid phages (Yagil *et al*. 1989). Experimental evidence using mutated and chimeric enzymes from bacteriophages λ and HK022 suggested a multistep process in which integrase specificity first broadens then narrows to permit co-evolution of the target site (Dorgai, Yagil and Weisberg 1995; Yagil, Dorgai and Weisberg 1995). Despite these efforts, the mechanisms underpinning integrase evolution remains somewhat murky. The special case of suicidal integrases presented in the following sections could shed some light on this fundamental process.

### Postmortem suicidal integrase excision activity… *in vivo*

After integration, the suicidal integrase gene is split in two inactive int(N) and int(C) pseudogenes potentially encoding the Int(N) N-terminal part and the Int(C) C-terminal part of the integrase respectively. The latter fragment carries the catalytic domain (Figs 2A and 3A). *In vitro* experiments concurred in demonstrating the inability of several truncated integrases to perform recombination reactions. The Int(N) and Int(C) moieties of IntSSV2 did not interact in solution in absence of DNA suggesting that they do not cooperate to assemble as an entire functional enzyme (Zhan, Zhou and Huang 2015). On its own, Int(C) could not form multimers since the N-terminal part of the integrase is responsible for multimerization (IntSSV1 dimerization and IntSSV2 tetramerization) (Zhan *et al*. 2012; Zhan, Zhou and Huang 2015). Contrastingly, *in vitro* recombination could be achieved with the truncated Int(C)SSV1 and Int(C)SSV2 albeit with a significantly reduced efficiency (Zhan *et al*. 2012; Zhan, Zhou and Huang 2015).

Several reports addressed the issue whether the expression of the Int(N) and Int(C) moieties could promote *in vivo* MGE excision in the absence of an intact integrase gene. First, the level of expression of the separate moieties was explored *in vivo*. In *S. solfataricus* P2, only the int(N) moiety is transcribed for the pXQ1 and XQ2 integrated elements (She *et al*. 2001; Jager *et al*. 2014) whereas no expression was detected for the Int(N) moiety nor for the complete integrase gene from integrated plasmid pSSVi in *S. solfataricus* (Ren, She and Huang 2013). In *T. nautili*, the IntpTN3 int(N) fragment lies downstream the integrase promoter and translation could be initiated at the original start codon while its int(C) moiety could be transcribed from the tRNA^Leu^ gene promoter (Cossu *et al*. 2017). For IntpTN3-related integrases, an in-frame start codon is often present near the beginning of int(C) suggesting that the catalytic part of the integrase could potentially be translated (our observation). So far, no consensus has emerged on the actual expression of the Int(N) and Int(C) moieties. Their level of expression might vary from one suicidal integrase to another resulting in various modes of excision control. In *S. solfataricus* P2 cells carrying the integrated plasmid pSSVi, episomal copies of this plasmid were barely detectable (Ren, She and Huang 2013). While infection of this strain with the related SSV2 virus accumulated free pSSVi plasmids, the increase was due to additional replication of rare episomes rather than to the excision of integrated copies (Ren, She and Huang 2013). However, SSV2 excision was shown to occur in the presence of the episomal MGE coding for the complete integrase (Fusco *et al*. 2015b). Similarly, the heterologous expression of plasmid pTN3 integrase in *T. kodakarensis* promoted excision of the related integrated element TKV4 (Cossu *et al*. 2017). Furthermore, TKV4 excision could be obtained in the same organism by supplying in*trans* a gene encoding inactive IntpTN3 Y428A, therefore suggesting that TKV4 Int(C) is effectively expressed (Cossu *et al*. 2017). This trans-complementation suggests that the Int(C) moiety might play a role in the excision of a mobile element by an exogenous integrase. For both SSV2 and pTN3, fragmented integrase and exogenous integrases were closely related. This excision catalysis by another MGE therefore depends on the widespread occurrence of closely related integrases in the population and in various MGEs that were observed for the pT26-2 family of integrases (Badel *et al*. 2020).

### Suicidal integrase maintenance, evolution and specificity switch

As mentioned above, it appears at first glance that integrated MGEs encoding suicidal integrases would remain permanently entrapped in the host chromosome. The inability of their fragmented integrase gene to encode an active enzyme would prevent the excision reaction and further propagation. One would therefore expect these particular MGE populations to decrease progressively and eventually disappear. Paradoxically, it was observed to the contrary that such integrated MGEs pervade entire populations in both *Crenarchaea* and *Euryarchaea* (Pauly *et al*. 2019; Badel *et al*. 2020). Several observations were instrumental in explaining this phenomenon. First, several related or unrelated MGEs can be integrated in the same chromosome at different loci (Badel *et al*. 2020). Second, different integrases present different integration sites and closely related archaeal integrases do not always target the same att site. For example, the classical integrases identified in pT26-2 related plasmids from *Methanococcales* can target tRNA^SerTGA^, tRNA^SerGCT^ or tRNA^LeuTAA^ (Badel *et al*. 2019). The suicidal integrases identified in pT26-2-related plasmids from *Thermococcales* can target 14 different tRNA genes (Badel *et al*. 2020). In both cases, the most probable evolutionary scenario involves an ancestral integrase with a single DNA substrate specificity followed by target diversification in the descendant lineages. Target switching is however restricted within the two classes of tRNA genes: those encoding tRNAs with a supplementary loop and those that encode tRNAs without such a loop (Badel *et al*. 2019). Furthermore, archaeal suicidal integrases harbor the translation of the att site within their protein sequence (Figs 2A and 3A) deepening the conundrum of specificity change. For them, a change in site specificity is mechanically reflected by a change in protein sequence. One could expect that such a change would compromise protein integrity, but it was shown on the contrary that the att site translation is quite variable in closely related sequences without obvious deleterious effect (Badel *et al*. 2020). Notably, length variations are compensated around the att site avoiding any frameshifts in the C-terminal region.

**Figure 13.**
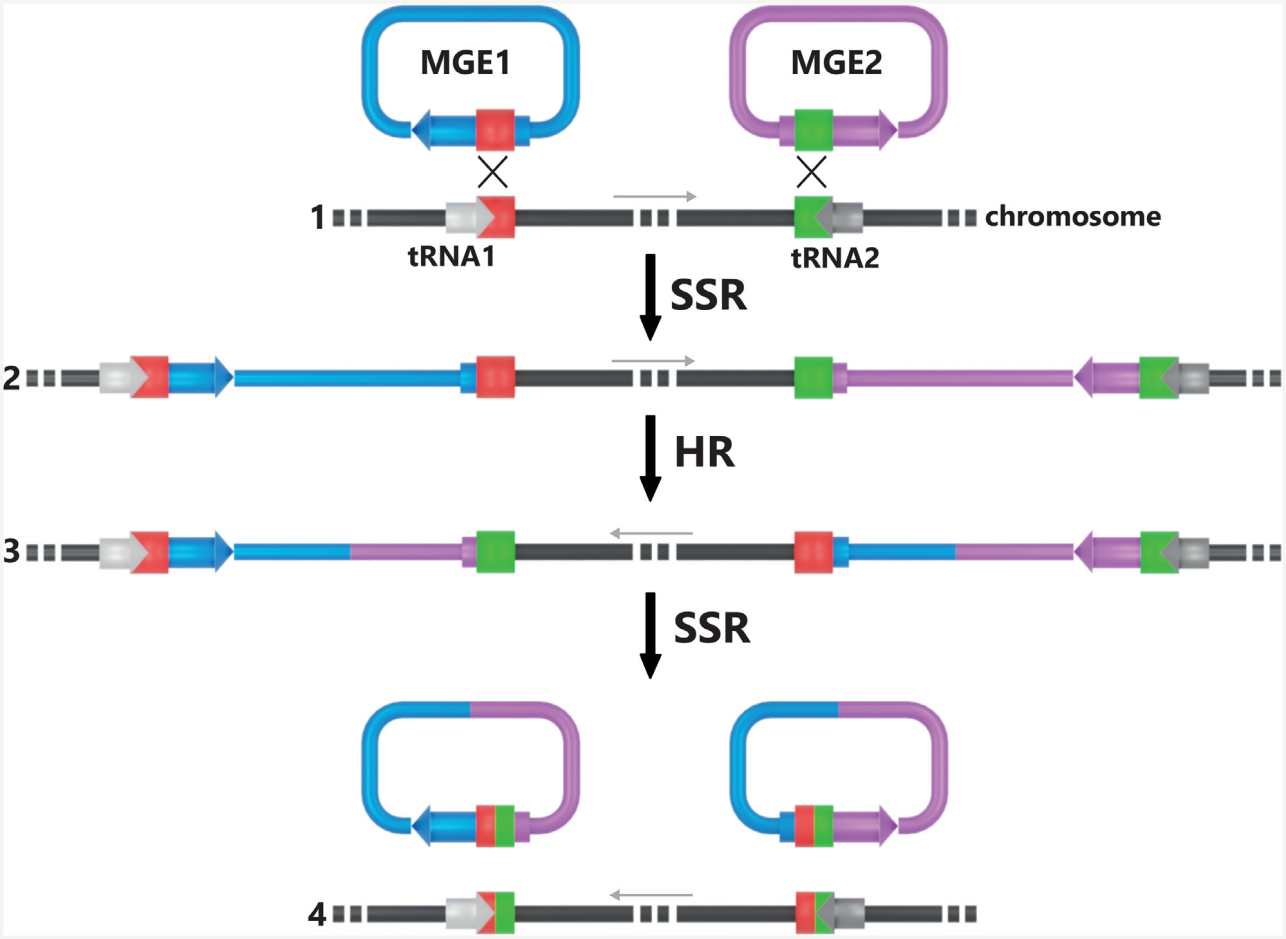
Suicidal integrase recombination. The integration by site-specific recombination (SSR) of multiple related MGE at different chromosomal locations and in inverted orientation **(A)** gives rise to homologous recombination (HR) between conserved MGE sequences **(B, C)**. Such a recombination has been observed in *T. kodakarensis* (Gehring *et al*. 2017). Recombinant integrated MGEs encoding hybrid integrases can then excise if a compatible integrase is provided by a superinfecting MGE **(D)**.

Accurate DNA and protein comparison of the genes and integrases belonging to the archaeal IntpT26-2 family underlined the differential evolution history of their Int(N), Int(C) and att components (Badel *et al*. 2020). It was argued that the integration of multiple elements sharing extensive sequence conservation could lead to homologous recombination (Redder *et al*. 2009; Gehring *et al*. 2017), generating chimeric integrase genes expressing active integrase (Badel *et al*. 2020). A model was proposed to explain the evolution and specificity switches in suicidal integrases. It is based on the observation of a large chromosomal inversion in a subset of the natural population of *T. kodakarensis* between TKV2 and TKV3, two related MGEs integrated in opposite orientation (Gehring *et al*. 2017). In this model, homologous inversion could generate two chimeric integrase genes by exchanging their int(N) and int(C) moieties and potentially novel attPs (Fig. 13) (Badel *et al*. 2020). Integrated MGEs encoding these chimeric suicidal integrases can be resurrected by superinfection of an incoming MGE with a compatible or more relaxed specificity and generate the observed variability (Cossu *et al*. 2017; Badel *et al*. 2020). This combinatorial mechanism does not only explain the pervasiveness of suicidal enzymes but also identifies the source of their variability.

## INTEGRASE-RELATED GENOME EVOLUTION

### Mobile genetic element modular evolution

Several reports underlined that bacterial MGE evolution proceeds mainly through module exchange (Botstein 1980; Oberto, Sloan and Weisberg 1994; Hendrix *et al*. 2000). The same rule applies to archaeal plasmids and viruses (Basta *et al*. 2009; Krupovic, Forterre and Bamford 2010; Iranzo *et al*. 2016). For example, the *Pyrococcus yayanosii* PYG1 integrated element shares a module with the MP integrated from *Thermococcus barophilus* element and another module with plasmid pTBMP1 of the same organism (Li *et al*. 2016). This mechanism of module exchange can be explained by homologous recombination involving several MGE integrated in tandem at the same chromosomal locus (Redder *et al*. 2009). Alternatively, homologous recombination between inverted modules of tandem integrated MGEs could lead to integrase-independent excision embarking portion of both integrated elements. MGE integration therefore facilitates this modular evolution. Additionally, integrases are directly involved in this process as several halophilic viruses were identified that encode tyrosine recombinases that seem implicated in viral DNA rearrangements (Rossler *et al*. 2004; Sencilo *et al*. 2013). One of the DNA rearrangements was involved in the generation of protein variants presenting various cell surface adhesion specificities (Klein *et al*. 2012).

### Horizontal gene transfer

Horizontal gene transfer (HGT) refers to the transmission of genetic information between individual organisms independently of direct progeny. HGT is recognized as a driving force of archaeal evolution (Wagner *et al*. 2017). Several successive steps are required for effective HGT: (i) DNA is transferred into the cell via transformation, membrane vesicle, viral infection, conjugation, cell fusion or other specialized cellular apparatus (Wagner *et al*. 2017). (ii) The foreign genetic information is incorporated into the host chromosome through homologous recombination or through site-specific integration if the incoming DNA carries an integration module. (iii) In the case of MGE-catalyzed integration, the DNA should be immobilized in the host chromosome to be effectively characterized as HGT. This can happen through integrase gene mutation or loss. A nonsense mutation present inside the integrase coding-sequence of the integrated element pST4 illustrated this case of HGT in *Sulfolobus* (She, Chen and Chen 2004). Similarly, mutations into the attL and attR sites would lead to MGE sequestration into the host chromosome. Integrated plasmids that lack detectable att sites were recently identified that might correspond to captured elements (Badel *et al*. 2019). Additionally, integration in secondary attachment sites, i.e. att sites with a mismatch, could prevent efficient MGE excision and lead to permanent acquisition of MGE genes by the host chromosome (She, Chen and Chen 2004).

If all functions can be transmitted by MGE-mediated HGT, the most studied was DNA replication. The archaea *Sulfolobus islandicus* and *Haloferax volcanii* possess several active chromosomal origins of replication, some of which were acquired from integrated MGE (Robinson and Bell 2007; Hawkins *et al*. 2013; Samson *et al*. 2013). At the archaeal domain scale, an exhaustive phylogenic analysis of all major replication components showed that chromosomal copies of several components (e.g. MCM, PCNA, PolB) probably arose from MGE integration (Raymann *et al*. 2014; Badel *et al*. 2019). Archaeal MGEs were also proposed to be implicated in the HGT of tRNA gene introns. Sugahara *et al*. (2012) proposed recombination between an intron-free tRNA gene attB and an intron-containing attP as a mechanism of intron acquisition in tRNAs where the MGE attP serves as intron vector between tRNAs and between cells.

### Chromosomal inversions

Among MGEs, transposable elements (TEs) are known to be frequently involved in generating inversions in the host chromosome (Eickbush and Furano 2002; Zivanovic *et al*. 2002; Redder and Garrett 2006; Darmon and Leach 2014; Weckselblatt and Rudd 2015; Vandecraen *et al*. 2017). Inversions proceed through homologous recombination between two paralogous integrated elements. Other integrated MGEs are also involved in similar processes. In *T. kodakarensis*, a large-scale inversion was identified that occurred through homologous recombination between the related integrated elements TKV2 and TKV3 (Gehring *et al*. 2017; Badel *et al*. 2019, 2020). Tyrosine recombinases are fundamental in that process because they catalyze the integration of paralogous MGE copies into the chromosome. Another unique mechanism was identified in archaea that led to chromosomal inversions. The tyrosine integrase from *Thermococcus nautili* plasmid pTN3 ‘catalyzes low sequence specificity recombination reactions with the same outcome as homologous recombination events’ between identical DNA segments as short as 104 bp (Cossu *et al*. 2017). This homologous recombination activity resulted in four large-scalet chromosomal inversions over the span of 66 generations in *T. nautili*. (Cossu *et al*. 2017). The broad occurrence of integrated MGEs carrying integrases similar to IntpTN3 is probably one of the major causes of the large chromosomal inversions observed in *Thermococcales*, especially in the *Thermococcus* genus where TEs are absent or rare (Cossu *et al*. 2015, 2017). Archaeal tyrosine recombinases are thus involved in chromosomal inversion either indirectly through the integration of multiple, recombinable, MGE copies or directly through the homologous recombination of original chromosomal segments. As a consequence of both mechanisms, chromosomes are largely disrupted in their otherwise conserved organization (Cossu *et al*. 2015). The fitness cost or benefice of such inversions is yet unknown.

## CONCLUSIONS AND FUTURE PERSPECTIVES

The combination of specific DNA binding and multimerization domains with a module capable to resect and exchange DNA strands gave rise to an enzyme widely adopted in all domains of life. With a remarkable efficiency and without the expense of energy, these recombinases allow the effortless host chromosome integration and excision of a number of MGEs. By resolving chromosomes dimers, tyrosine enzymes also provide the opportunity to correct flaws resulting from circular chromosomal replication in bacteria, archaea and large bacteriophages.

In the last three decades since the identification of the first integrative element in Archaea, the study of tyrosine recombinases revealed common aspects for the three domains of life, such as allowing both MGE integration and excision and their propensity to target tRNA genes in the host genome. The primary sequences of archaeal, bacterial and eukaryal tyrosine recombinases are highly diverse, precluding the use of phylogeny to assess relationships between these enzymes. With a network analysis, we generated a robust classification of archaeal integrases while confirming and extending previous comparisons based on Pfam protein domains. Going further, a global evolutionary analysis of all tyrosine recombinases from archaeal, bacterial and eukaryotes could be undertaken. It would evidence potential transfers between the three domains and would shed some light on the origin of tyrosine recombinases. On other aspects, archaeal integrases differ from bacterial and eukaryal integrases. They do not require essential helper or directionality factors, and hyperthermophilic archaea have developed a particular suicidal integration system where the MGE target site is carried within the integrase gene.

Archaeal integrases have now proved to be important models for understanding tyrosine recombinases. The study of suicidal integrases found exclusively in the archaeal domain provided important clues on the evolution of these enzymes. Additionally, the study of a new tyrosine recombinase led to discovery of an integrase family capable of the dual activity of site-specific and homologous recombination. Both examples warrant the further investigation of archaeal tyrosine recombinases, including the new integrase families identified in our network analysis.

On many other aspects, archaeal integrases still have much to reveal. Archaeal MGE lysogeny was never studied in detail despite the obvious differences with the canonical phage λ lysogeny. Such approaches would lead to a better understanding of the dynamics of MGE integration and excision in natural archaeal communities. The study of archaeal MGE would also help determine whether integration is preferentially implemented in certain environmental or genetic conditions.

Recent advances in the crystal structure resolution of several archaeal tyrosine recombinases successfully demonstrated similarities of the catalytic domain with other known bacterial enzymes. However, the modalities by which archaeal recombinases interact with their DNA substrate remain to be explored and could be approached by solving the complete structure of protein–DNA complexes by co-crystallization or cryo-electron microscopy.

The development of large-scale genome sequencing is bound to improve the knowledge of genome dynamics and might emphasize the already acknowledged importance of MGE integration in this process. Further studies should also investigate the multiple integration of archaeal MGEs and how they shape the genome evolution and diversity of their hosts.

## DATA AVAILABILITY

The protein sequence data files used in the archaeal network analysis are provided in Fasta format at https://archaea.i2bc.paris-saclay.fr/Archaea_Int.zip.

## Supplementary Material

fuab004_Supplemental_Files
