# Supplementary figures and images for: Archaeal tyrosine recombinases
Source: FEMS Microbiol Rev. 2021 Feb 1;45(4):fuab004. doi: 10.1093/femsre/fuab004 (PMC8371274; doi:10.1093/femsre/fuab004)

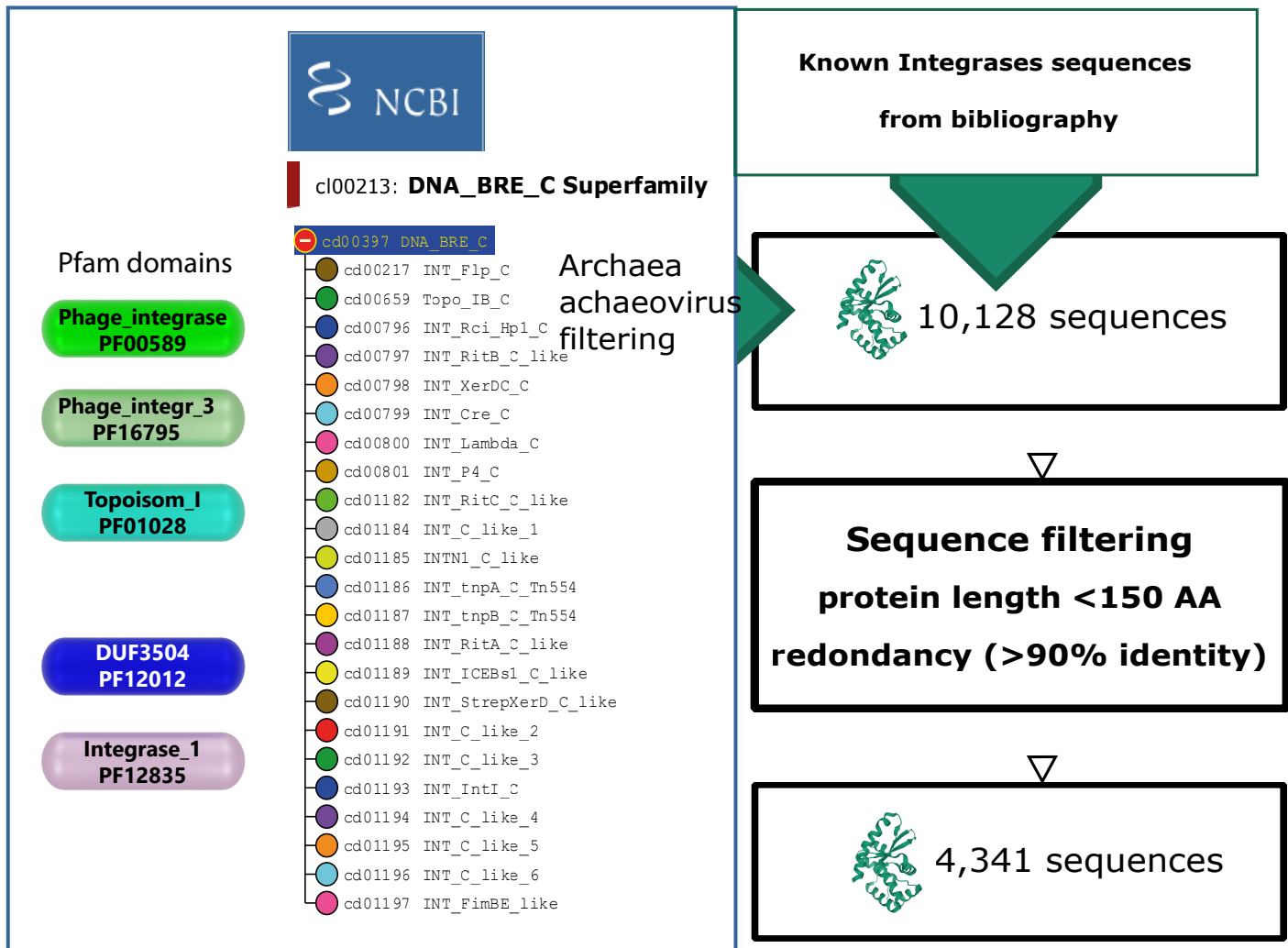

Supplement: fuab004_Supplemental_Files [file fuab004_supplemental_files.zip › Suppl_Figure_1_Pipeline.pdf]

**Suppl. Figure 2**

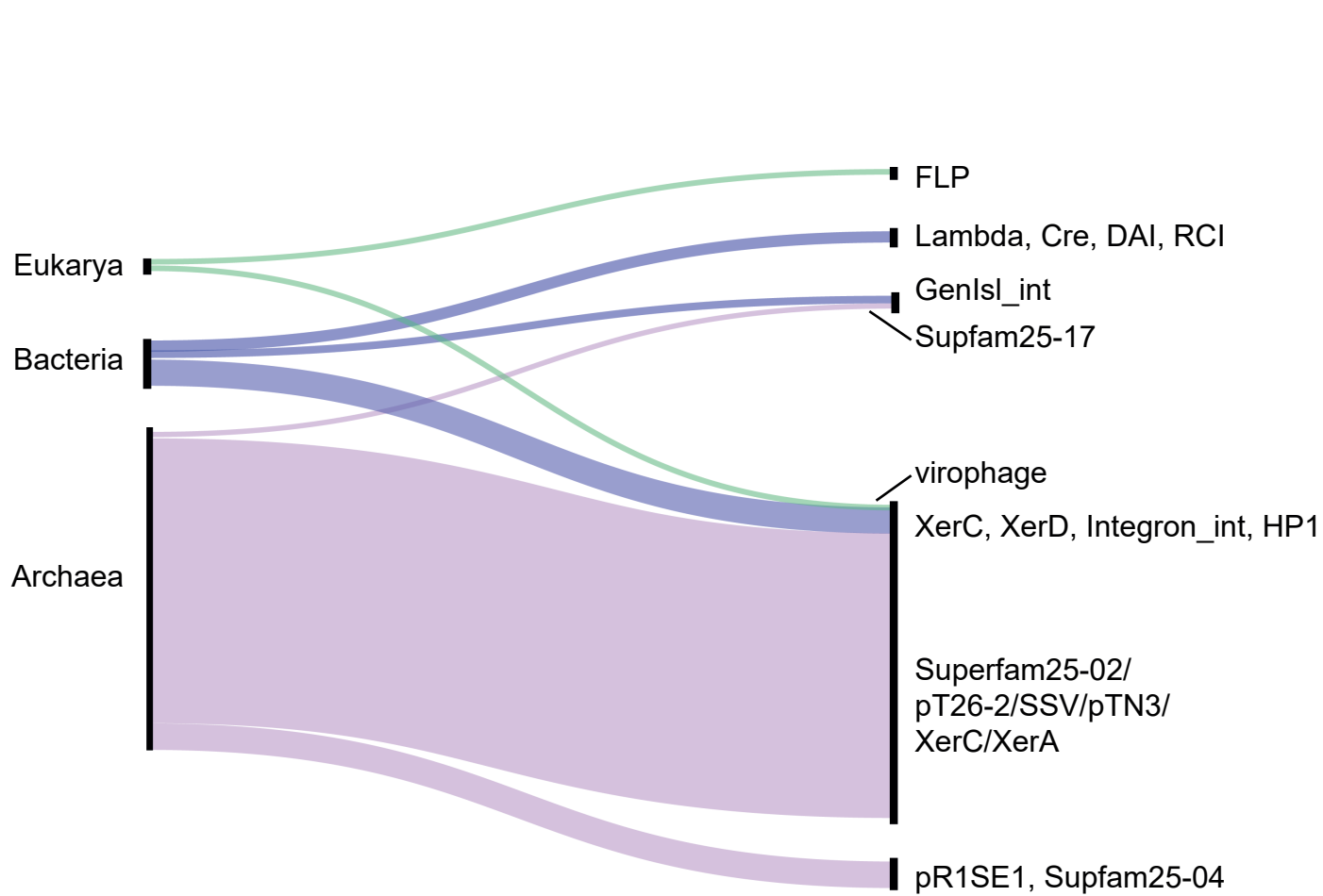

Supplement: fuab004_Supplemental_Files [file fuab004_supplemental_files.zip › Suppl_Figure_2_Alluvial_3_domains.pdf]
